# Hydrogen extends *Caenorhabditis elegans* longevity by reducing reactive oxygen species

**DOI:** 10.1371/journal.pone.0231972

**Published:** 2020-04-22

**Authors:** Miao Zhang, Zhihui Li, Dawen Gao, Wenjing Gong, Yan Gao, Chenggang Zhang

**Affiliations:** Institute of Radiation Medicine, Academy of Military Medical Sciences, Academy of Military Sciences, Military Cognitive and Mental Health Research Center of PLA, Beijing, China; Inha University, REPUBLIC OF KOREA

## Abstract

At present, a large number of studies have reported that hydrogen has antioxidant functions and prevents oxidative stress damage. However, it is not clear whether hydrogen can prolong longevity based on these effects. Therefore, we studied and explored the antiaging potential of exogenous hydrogen and its ability to extend longevity using *Caenorhabditis elegans* (*C*. *elegans*) as an animal model. Our results showed that the lifespans of the N2, *sod-3* and *sod-5* mutant strains were extended by approximately 22.7%, 9.5%, and 8.7%, respectively, after hydrogen treatment, but hydrogen had no effect on the lifespans of the *daf-2* and *daf-16* mutant strains. Meanwhile, the level of reactive oxygen species (ROS) in the hydrogen treatment group was significantly lower than that in the control group. At the transcript level, the expression of *age-1* and *let-363* was obviously decreased, while the expression of *ins-18* was increased at the same time point (14 d). Compared with the control group, paraquat (PQ) could reduce the lifespan of the N2 and *sod-5* mutant strains. Importantly, the longevity of these mutant strains recovered to normal levels when the animals were treated with exogenous hydrogen. According to these results, the lifespan of *C*. *elegans* is closely related to oxidative stress and can be significantly prolonged by reducing oxidative stress damage. Taken together, our data showed that hydrogen is a valuable antioxidant that can significantly reduce the body’s ROS levels and extend the lifespan of *C*. *elegans*. This study also laid a foundation for the subsequent application of hydrogen in antiaging studies.

## Introduction

It is well known that hydrogen can effectively scavenge free radicals *in vivo* or *in vitro* and exhibit valuable antioxidant activity[1,2]. Under stress such as ischemia or hypoxia in the brain, heart and other vital organs and tissues, immune cells release a large amount of reactive oxygen species (ROS), while hydrogen can selectively neutralize hydroxyl radicals and peroxynitrites, which are related to the activation of the Nrf2 signaling pathway[1,3]. Hydrogen-rich saline (HRS) can also reduce the damage to important organs, tissues and cells caused by oxidative stress[4]. In general, hydrogen has two advantages compared with other antioxidants, such as vitamin A and vitamin C. First, hydrogen can selectively neutralize hydroxyl radicals and nitrite anions[5]. Second, it can quickly reach the area in danger regardless of cellular barriers[6]. The fact that antioxidants have limited therapeutic success may be because most antioxidants cannot reach specific ROS-abundant regions[7]. Thus, hydrogen can be used as an effective antioxidant therapy owing to its ability to diffuse rapidly across cellular membranes, because it can reach and react with cytotoxic ROS and protect against oxidative damage[1].

Aging is a complex process of natural degradation. During the aging process, the functions of the body tissues and organs degrade, and immunity is reduced, resulting in a decrease in the body’s ability to adapt and fight infection and eventually leading to the end of life[5]. At present, there are several theories focusing on the mechanisms of aging, such as free radical damage, caloric restriction, and telomere senescence. The free radical aging hypothesis suggests that aging is caused by oxidative damage to cells, tissues, *etc*.[5]. In addition, oxidative stress has been proposed to be one of the major causes of aging and has been implicated in the pathogenesis of many diseases[8]. Therefore, a certain concentration of antioxidants will contribute to the longevity of organisms. For example, Tsai Tai (*Brassica chinensis*) extracts significantly increased resistance against paraquat (PQ)-induced oxidative stress, with an increase in survival rates from 15% to 28% compared with controls; pakchoi ethanolic hydrochloric acid extract increased the survival rate of *C*. *elegans* under PQ stress[9]. It is thus clear that aging is closely related to oxidative stress.

Because oxidative stress is caused by excessive ROS, which can be produced from endogenous or exogenous sources[10], the expression of ROS has been used to estimate and identify oxidative stress damage. ROS are a product of aerobic tissue energy metabolism. When ROS are produced in excess or the endogenous antioxidant capacity is weakened, homeostasis is broken, and oxidative stress occurs, resulting in body injury. For example, a number of approaches have been used to determine sensitivity to oxidative stress in *C*. *elegans*, including exposure to PQ, juglone, t-BOOH, arsenite, H_2_O_2_, or hyperbaric oxygen[11]. Therefore, PQ was used to induce an endogenous oxidative stress model in *C*. *elegans* in this paper[12]. In addition, there are known signaling pathways that affect aging, such as the insulin/insulin-like growth factor-1 signaling pathway, sirtuin family signaling pathways and the target of rapamycin (TOR) signaling pathway, which have been studied in various models, such as *C*. *elegans*, yeast, fruit flies, and mammals[13]. Thus, we investigated and estimated the ability of hydrogen to counteract aging and extend longevity by measuring the ROS level and insulin/insulin-like growth factor-1 signaling pathway after hydrogen treatment with or without PQ.

## Materials and methods

### Chemical and strains

Wild-type *C*. *elegans* (N2), *daf-2* (e1370), *daf-16* (mu86) and *sod-3* (gk235) were provided by the Caenorhabditis Genetics Center (CGC). The mutants *sod-5* (tm1146) and *Escherichia coli* OP50 (Streptomycin^+^) were presented by Professor Mitani S of Tokyo Women’s Medical University. PQ and CM-H2DCFDA probes were purchased from Sigma Company (USA). RNA extraction kits and DNase were purchased from Omega (USA); reverse transcription kits were purchased from Toyobo (Japan); ExTaq enzyme, DNA Marker DL2000, and dNTP Mixture were purchased from TaKaRa (Japan); agarose was purchased from Gene (Hong Kong, China); and GoldView^™^ nucleic acid dye was purchased from Solarbio (China).

All strains were maintained and grown on NGM plates seeded with *E*. *coli* OP50. NGM plates containing PQ were equilibrated overnight before use. Hydrogen was generated by a hydrogen gas generator SHC-300 (Saikesaisi HW Energy, Shandong, China). The concentration of hydrogen gas was measured by the hydrogen detector HD-P900X-H2 (Jinan Handa Electronics Technology Co. Ltd., Shandong, China).

### Experimental diagram

All worms were cultured on fresh NGM plates, fed adequately, and subjected to life analysis at 20 °C. The day on which the L1 larvae were transferred to NGM plates was defined as day 0. From day 0, the worms were treated with hydrogen for 4 h per day until the end of their life span (S1 Fig). A body length assay was carried out on days 1, 3 and 5; a reproduction assay was carried out on day 2; a ROS assay was carried out on days 7 and 14; a lifespan assay was carried out on day 10; and a gene expression assay was carried out on day 15 (S2 Fig).

### Lifespan assay

All worms were cultured on fresh NGM plates, fed adequately, and subjected to life analysis at 20 °C. The day on which the L1 larvae were transferred to NGM plates was defined as day 0. When the worms entered the adult stage and began to lay eggs, they were transferred to fresh plates every day. Starting on day 10, the survival, death and loss of *C*. *elegans* were consecutively recorded every day. The worms that did not respond to a mechanical stimulus were scored as dead. When the worms crawled off the plate, displayed extruded internal organs, died from hatching progeny inside the uterus or had a bag-of-worms, they were not counted in the death number. About ten nematodes in each group were not counted in death number. The experiment was repeated at least three times.

### Head swinging assay

The worms incubated at 20 °C for 15 d were picked up and transferred to sterile NGM medium under a stereomicroscope. After free movement for 30 s, the frequency of head swings in 1 min was counted. The head swings were recorded once per worm, and 10 worms from each group were counted. The experiment was repeated at least three times.

### Reproduction assay

After synchronization, the worms were cultured for 1 d at 20°C. Nine worms from each group were placed in NGM plates (3 cm in diameter) containing no PQ or 0.5 mM PQ[14]. Three worms were placed per plate for a total of three plates per group. The hydrogen group underwent daily exposure for 4 hours, and the control group was cultured normally. The number of eggs laid by the worms was counted every day until no more progeny were generated, and then, the egg number was summed to determine brood size [15]. Each experiment was repeated at least three times.

### ROS assay

First, adult worms were washed and collected from the plates with M9 buffer and were subsequently cleaned three times. Then, the worms were transferred to 5 ml Hank’s solution containing 10 μM CM-H2DCFDA probe and incubated at 20°C for 30 minutes. Next, the worms were washed three times with M9 buffer and then picked up on an agarose gel pad with 20 μl of 5 mM levamisole hydrochloride, covered with a cover glass. Finally, the worms were observed and imaged by a fluorescence microscope with a microscope system (excitation wavelength 488 nm, emission wavelength 510 nm). The integrated optical density (IOD) of each nematode was quantified by Image-Pro Plus 6.0 software (Media Cybernetics, Inc., 8484 Georgia Avenue, Silver Spring, Maryland 20910, USA). More than 10 worms were measured for each experiment. The experiments were repeated at least three times.

### Gene expression assay

Approximately 300 synchronized young adult worms were transferred to NGM plates (9-cm diameter) with or without hydrogen gas, and cultured at 20 °C for 15 d. Total RNA was extracted using an RNA Kit (Omega) and converted to cDNA using a High Capacity cDNA Reverse Transcription Kit (Toyobo). The cDNA samples were added to the following primers for PCR. The relative expression levels of the genes were assessed using an agarose gel electrophoresis apparatus (Bio-Rad, USA) and the image analysis software Image-Pro Plus (IPP) 6.0. The primers used are listed in Table 1. The experiment was repeated at least three times.

**Table 1:** Primer information of the genes.

Gene name	Primer sequence
*gpd-1*	Fwd: 5’-ATGGGGATCAGTCAAAGCCG-3’
	Rvs: 5’-TCGACTGTCTTCTGGGTTGC-3’
*age-1*	Fwd: 5’-TGCTCTCCGAACTCGCATTT-3’
	Rvs: 5’-GCTCGTCACGCAGTTTCATC-3’
*let-363*	Fwd: 5’-ACTTGGTTCACTCGTCGGTC-3’
	Rvs: 5’-AATTGCGCAACGAACAAGCT-3’
*ins-18*	Fwd: 5’-ACGCATGAAAATGTGCCCAC-3’
	Rvs: 5’-AATTGGGGCACAGTAGGCAA-3’

### Body length assay

The worms that grew to the L1 phase after the synchronization treatment were transferred to PQ (0.5 mM) medium [14]. The hydrogen group was exposed for 4 h per day. On days 1, 3 and 5 after the end of hydrogen exposure, the body length of the worms was observed under a stereomicroscope. Image acquisition was performed using NIS-Elements F 40000 software, and the images were analyzed with Image-Pro Plus 6.0 software. The experiment was repeated at least three times.

### Survival curve after PQ treatment

When the worms grew to L1 stage after synchronization, they were transferred to different NGM medium and exposed to hydrogen for 4 hours per day. When the worms grew to the adult stage, they began to lay eggs and were transferred to new medium every day. After the hydrogen treatment on day 9, 200 worms in each group were transferred to PQ medium under a microscope (100 in 0.5 mM and 100 in 5 mM PQ medium). The hydrogen group continued to receive hydrogen gas treatment every day, while the control group did not. The survival, death and loss counts of the worms were recorded starting on day 10. The experiment was repeated at least three times.

### Statistical analyses

Statistical analyses of lifespan were performed using the GraphPad Prism 6 package. Kaplan-Meier lifespan analysis was performed, and *p* values were calculated using the log-rank test. IPP 6.0 software was used to analyze the gel and fluorescence images. SPSS 16.0 software was used for statistical analysis of the other results. These results are expressed as the mean±SD, and *p* values were calculated by a two-tailed *t-*test analysis. *p<0.05 was considered a significant difference, and **p<0.01 was considered a very significant difference.

## Results

### Hydrogen can extend the lifespan of *C*. *elegans*

**Fig 1:**
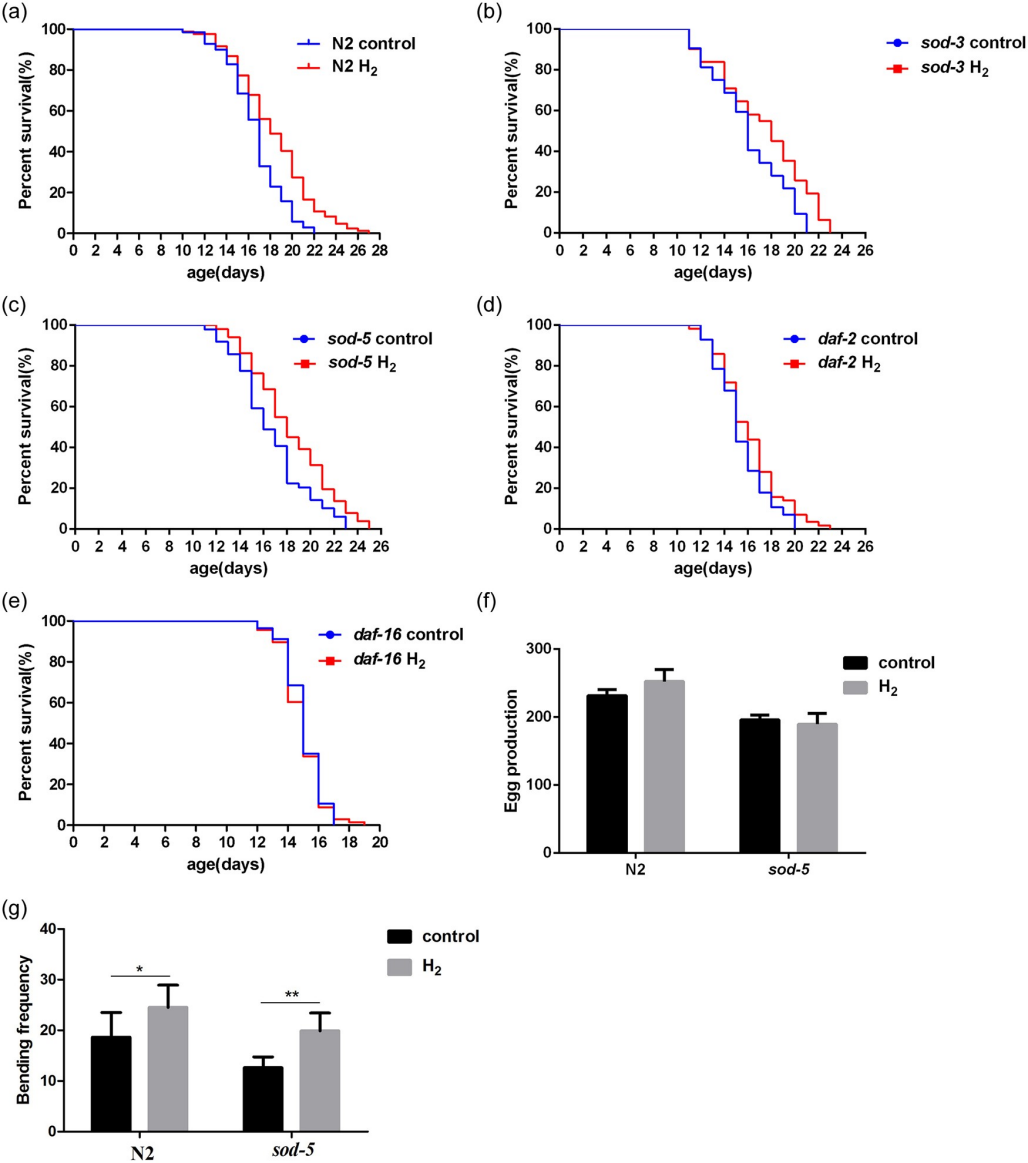
Effects of hydrogen on the lifespan, reproduction and motor behavior of *C*. *elegans*. (A-E) Representative Kaplan-Meier survival curves of N2, *daf-2*, *daf-16*, *sod-3* and *sod-5* nematodes after hydrogen treatment (>60 animals per group). (F): Spawning of N2 and *sod-5* nematodes with or without hydrogen treatment. (G): N2 and *sod-5* nematode head swing frequencies with or without hydrogen treatment. (F) and (G): Data are shown as the mean±SD of three independent experiments. *p<0.*05*; ** *p<0*.*01*.

Hydrogen was produced under laboratory conditions. When the volume of hydrogen at the outlet was stabilized above 10000 ppm (Table 2), the investigation was started. The results showed that hydrogen could significantly extend the age of wild-type N2 worms (*p = 0*.*0001*) (Fig 1A). We further examined the effects of hydrogen on the *sod-3*, *sod-5*, *daf-2* and *daf-16* mutant strains. Our results showed that hydrogen significantly prolonged the lifespan of the *sod-3* and *sod-5* mutant strains (*p = 0*.*0427*, *p = 0*.*0160*) (Fig 1B and 1C) but not the lifespan of the *daf-2* and *daf-16* mutant strains (*p = 0*.*1598*, *p = 0*.*7801*) (Fig 1D and 1E).

**Table 2:** Determination of hydrogen concentration.

Number of tests	Hydrogen concentration (ppm)			
	First measure-ment	Second measure-ment	Third measure-ment	Mean±SD
1	13528	9988	8466	10660.67±2597.17
2	9013	11712	10927	10550.67±1388.30
3	12775	10955	9582	11104.00±1601.71
4	11426	8830	10846	10367.33±1362.59

Previous studies have shown that reproduction is a proximal cause of senescence because within a generation, reducing reproductive activity can extend life span[16]. However, our data showed that hydrogen does not reduce the number of spawnings per day or the total number of offspring per worm (S3 Fig and Fig 1F). The head bending frequency was also improved after hydrogen treatment (Fig 1G). It is thus clear that hydrogen has no effect on spawning number, but can significantly improve the movement ability of *C*. *elegans*.

### Hydrogen can downregulate active oxygen levels

**Fig 2:**
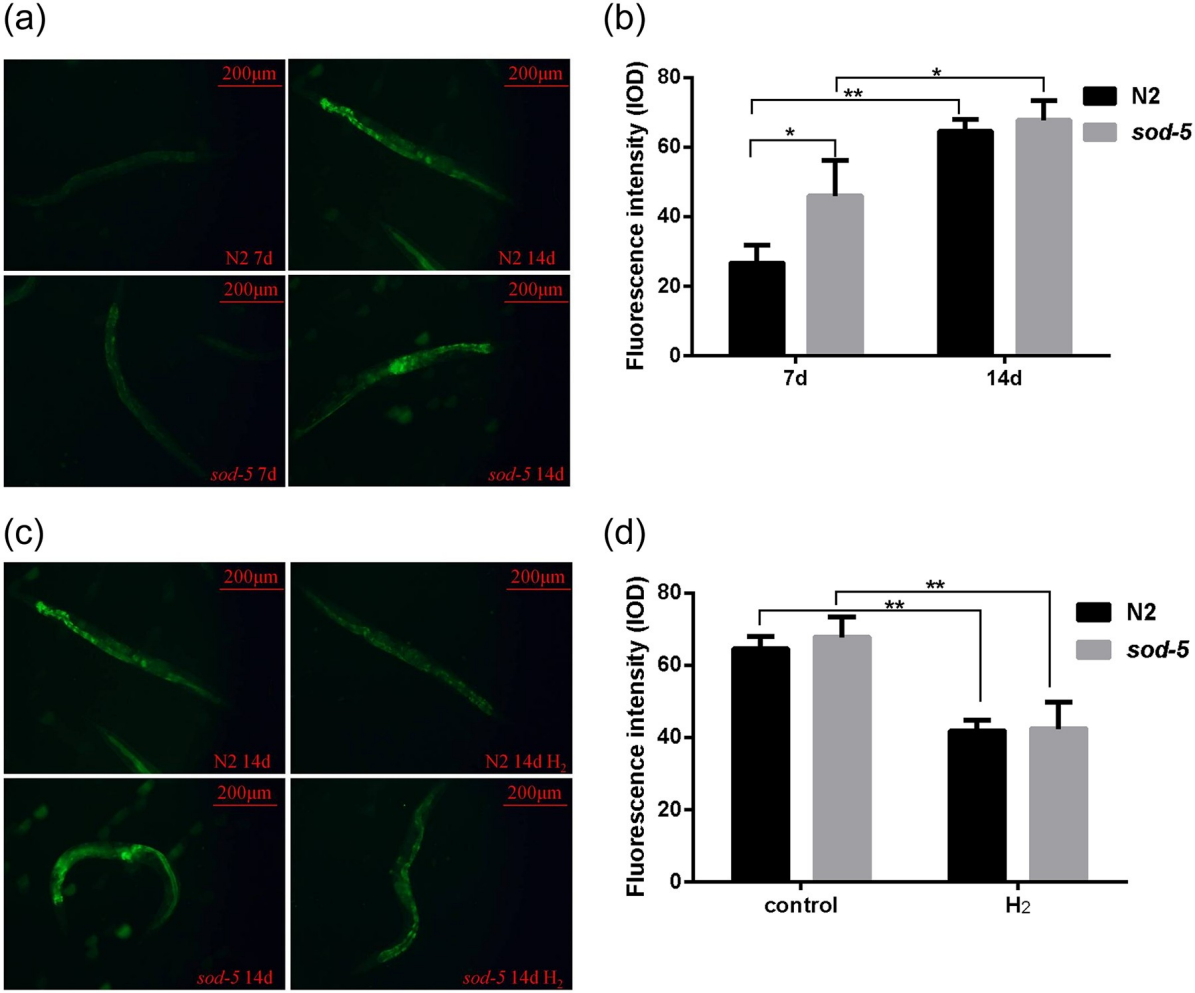
Effects of hydrogen on ROS levels in *C*. *elegans*. (A) and (C): Corresponding fluorescent images captured by the Image Xpress Micro System. (B): ROS levels of N2 and *sod-5* nematodes (days 7 and 14). (C): Effects of hydrogen on ROS levels of N2 and *sod-5* nematodes (day 14). Fluorescence intensity was analyzed using Image-Pro Plus 6.0, and the data are shown as the mean ± SD of three independent experiments. *p<0.*05*; ** *p<0*.*01*.

Because active oxygen is an indispensable oxidizing substance in life activities and ROS levels are an important indicator of oxidative stress in the body, we used fluorescent staining to analyze the change in ROS levels in worms to reveal the effect of hydrogen on *C*. *elegans*. Our results showed that the ROS levels were different at different time points in worms (Fig 2A and 2B). Compared with day 7, the ROS level on day 14 was much higher in the N2 and *sod-5* mutant strains. Meanwhile, the ROS level of the *sod-5* mutant strain was much higher than that of the N2 strain at the same time point. After hydrogen treatment, the ROS levels were significantly reduced in the N2 and *sod-5* mutant strains (Fig 2C and 2D). Therefore, to a certain extent, hydrogen as a supplemental exogenous antioxidant can neutralize the active oxygen from organisms and reduce ROS levels or oxidative damage.

### Changes in the expression of lifespan-related genes after hydrogen treatment

**Fig 3:**
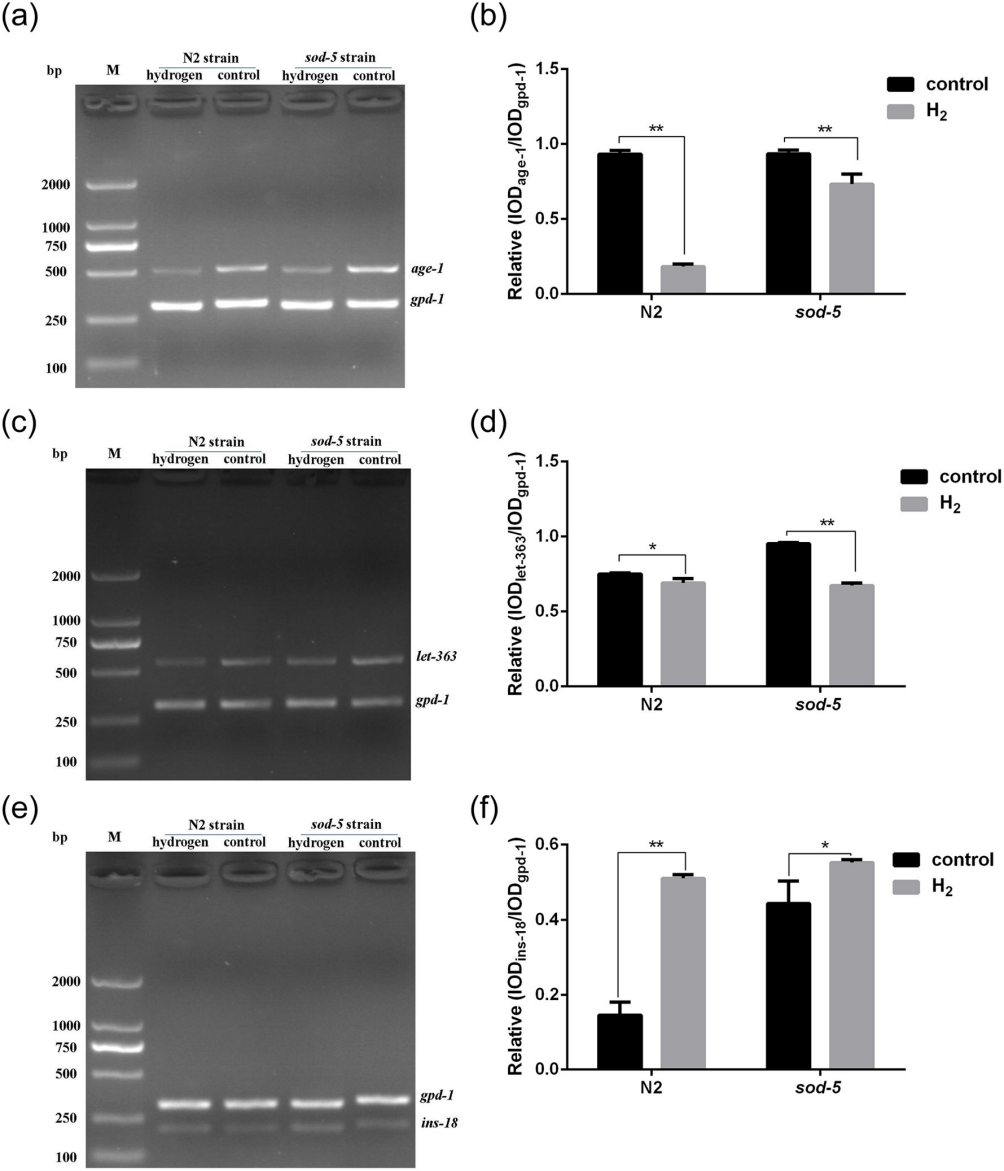
Expression of several genes in *C*. *elegans* after hydrogen treatment. (A), (C) and (E): A gel image of the PCR product was collected by a gel imager. (B), (D), and (F): The integrated optical density (IOD) values of each gene fragment were analyzed by Image-Pro Plus 6.0. The ratio of IOD of the target gene to the internal reference gene *gpd-1* was used as a measure of the level of expression of the target gene. Data are shown as the mean ± SD of three independent experiments. * *p<0*.*05*; ** *p<0*.*01*.

It has been reported that the expression of the *age-1*, *let-363* and *ins-18* genes is closely related to longevity[17–19]. For example, the physiological function of INS-18 was first examined by gene disruption and overexpression, and INS-18 was identified as a DAF-2 antagonist required for larval diapause and longevity[17]. The *age-l(hx546)* mutation prolongs the life of males and plays some role in male physiology or metabolism as well[18]. Therefore, we examined the expression of *age-1*, *let-363* and *ins-18* genes in nematodes of the same age. The results showed that the expression of *age-1* and *let-363* genes was downregulated (Fig 3A–3D), and the expression of *ins-18* gene was upregulated after hydrogen treatment (Fig 3E and 3F).

### Hydrogen protects against PQ-induced damage

**Fig 4:**
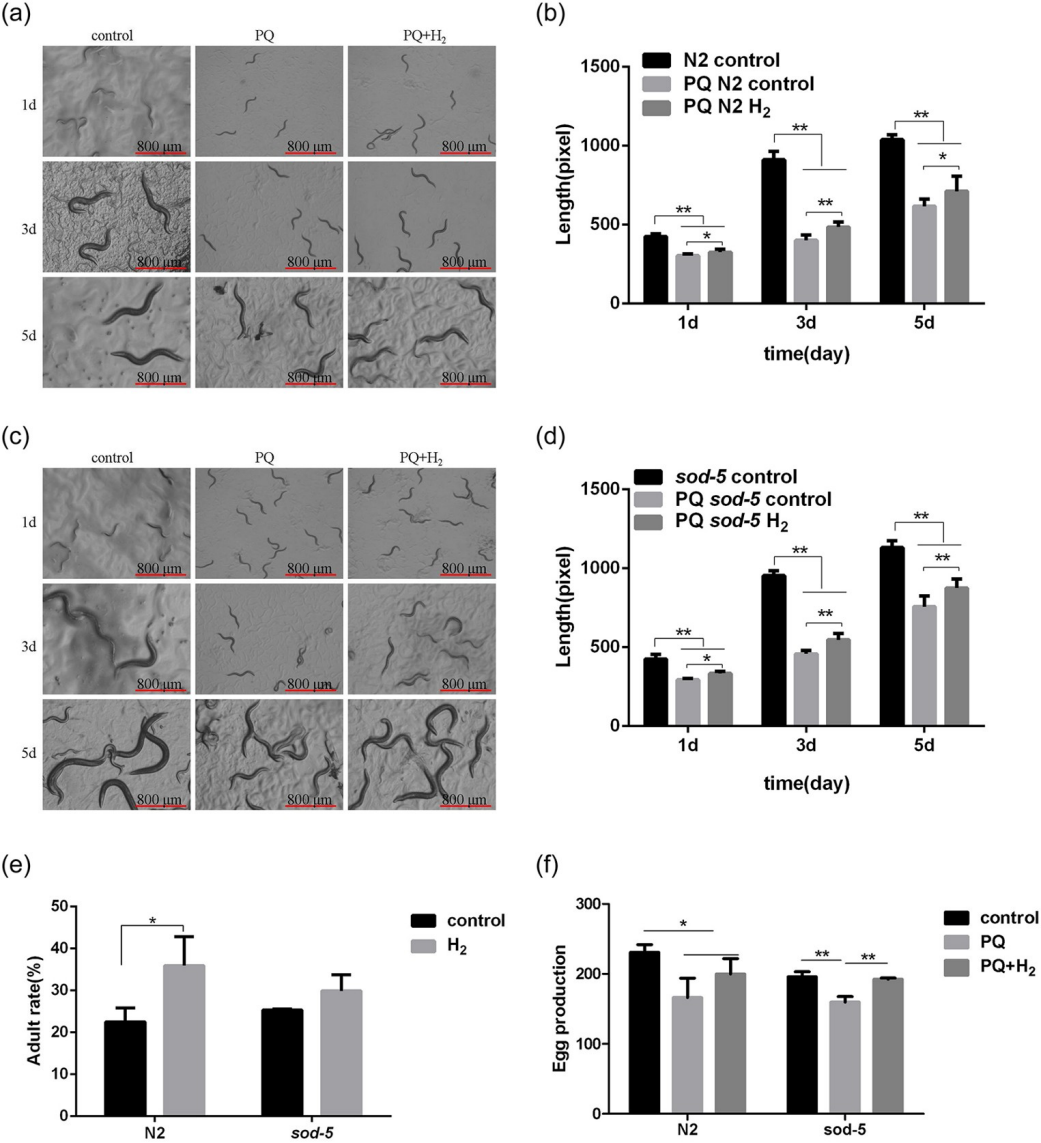
Effect of hydrogen on the growth and development of *C*. *elegans* after PQ-induced damage. (A) and (C) Body length changes of N2 and *sod-5* nematodes with or without PQ after hydrogen treatment. (B) and (D) The body length of each nematode was calculated using the image analysis software IPP 6.0 (>10 animals per group). (E) Adult rates of N2 and *sod-5* nematodes with or without PQ after hydrogen treatment. (F) Spawning of N2 and *sod-5* nematodes with or without PQ conditions after hydrogen treatment. (B), (D), (E) and (F): Data are shown as the mean ± SD. The concentration of PQ was 0.5 mM. *p<0.*05*; ** *p<0*.*01*.

When the wild-type strains were cultivated on 0.5 mM PQ, 0.5 mM PQ+hydrogen and the control condition after day 1, day 3 and day 5, the data showed that the body length of the PQ and PQ+hydrogen groups was significantly smaller than that of the control group. The body length recovered in the PQ+hydrogen group compared with the PQ group but was still lower than that in the control group (Fig 4A and 4B). The same phenomenon was also observed in the *sod-5* mutant strain (Fig 4C and 4D).

Because the adult rate can reflect the degree of PQ-induced damage, it becomes an important indicator for evaluating PQ-induced damage. Spawning is also an important indicator of growth and development. In this study, we found that the adult rate was less than 30% in the PQ group (Fig 4E), and the number of eggs was also much lower than that in the control group (Fig 4F). As expected, both the adult rate and the number of eggs were obviously increased after hydrogen administration (Fig 4E and 4F). However, except for the number of N2 nematodes spawning on the fourth day, the number of N2 and *sod-5* nematodes spawning per day did not significantly change after PQ-induced damage (S4 and S5 Figs).

**Fig 5:**
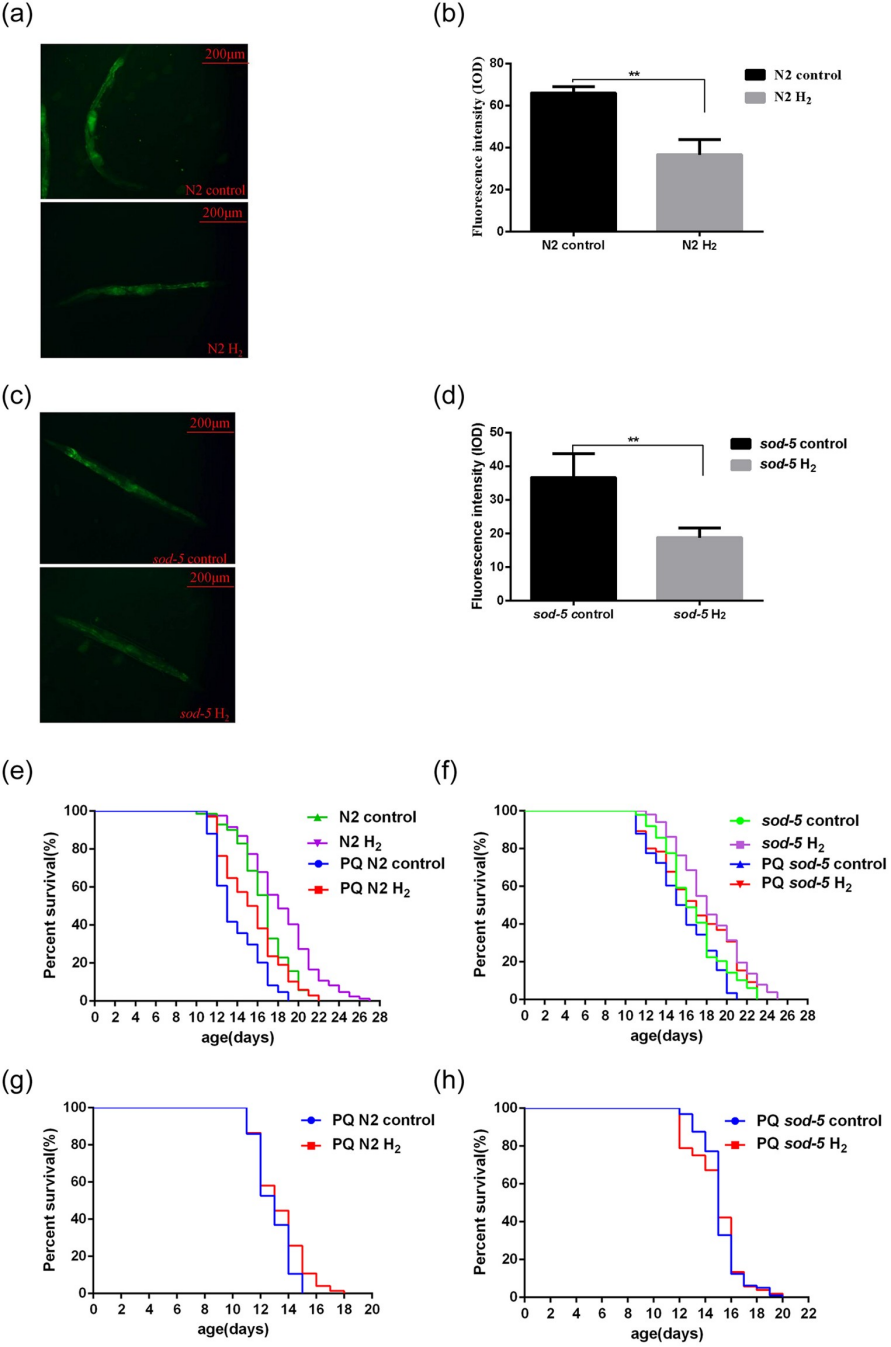
Effect of hydrogen on ROS levels and longevity of *C*. *elegans* under PQ-induced insults. (A) and (C) Corresponding fluorescent images captured by the ImageXpress Micro System. (A) Effect of hydrogen on ROS levels of the N2 strain under PQ treatment. (C) Effect of hydrogen on ROS levels of the *sod-5* mutant strain under PQ treatment. (B) and (D) Fluorescence intensity was analyzed using Image-Pro Plus 6.0, and the data are shown as the mean±SD. (E-H) Changes in longevity under PQ conditions with or without hydrogen treatment. Representative Kaplan-Meier survival curves are shown from three independent experiments (>60 animals per group). (A-F) The concentration of PQ was 0.5 mM; (G-H) The concentration of PQ was 5 mM. *p<0.*05*; **p<0.*01*.

To study whether hydrogen affects the lifespan of *C*. *elegans* through altering ROS, we further examined the changes in ROS levels and lifespan after PQ treatment. Our results showed that the ROS levels of the N2 and *sod-5* strains in the hydrogen group were lower than those in the PQ treatment group (*p = 0*.*0003*, *p = 0*.*0100*) (Fig 5A–5D). Hydrogen may not only prolong the lifespan of N2 and *sod-5* strains under 0.5 mM PQ conditions and restore their longevity to normal (*p = 0*.*0001*, *p = 0*.*0002*) (Fig 5E and 5F) but also extend the lifespan of N2 strains under 5 mM PQ stress conditions (*p = 0*.*0412*) (Fig 5G). However, under 5 mM PQ conditions, the prolongation effect was not obvious after hydrogen treatment (*p = 0*.*9055*) (Fig 5H). Therefore, hydrogen can not only prolong normal life but also prolong life under oxidative stress, although it has no obvious effect under serious oxidative damage.

## Discussion

The free radical aging theory, which is also called the aging oxidative stress theory, states that aging is caused by normal oxidative metabolism by-products such as ROS[5,20]. Normally, the antioxidant defense system eliminates ROS, and living organisms are protected from oxidative stress. Therefore, weakening of the antioxidant defense system, which may be caused by several factors, such as aging, will lead to excess oxidative stress and senescence[10]. The detection of hydrogen peroxide (H_2_O_2_) further suggests that aging is caused by excess ROS[21]. In *C*. *elegans*, genes such as *sod-1*, *sod-4* and *sod-5* encode Cu/Zn-SODs, and *sod-2* and *sod-3* encode Fe/Mn-SODs. Therefore, mutations in *sod* family genes may impact defense against oxidative stress[22]. In this study, we found that older nematodes have higher ROS levels. Interestingly, after hydrogen treatment, the ROS levels were significantly decreased, and hydrogen could significantly extend the lifespans of the *sod-3*, *sod-5* and N2 strains. In addition, aging is regulated by a variety of pathways, such as the insulin signaling pathway, the rapamycin target signaling pathway, and the caloric restriction pathway[23–25]. However, our results showed that the lifespans of the *daf-2* and *daf-16* strains were not affected after hydrogen treatment. Based on these data and previous reports that hydrogen is a valuable antioxidant *in vitro*, lifespan extension by hydrogen is mostly related to ROS levels. It seemed that exogenous hydrogen does not act through the insulin signaling pathway to produce its antiaging effects, which may result from a direct reaction with ROS *in vivo*.

Studies in recent years have shown that germ cells may accelerate aging by releasing signals, and loss of these signals may extend lifespan[16,26]. Although hydrogen could significantly prolong the lifespan of N2 and *sod-5* mutant strains, it had no effect on the number of spawning events, while the body bending and head swinging frequency of nematodes were significantly increased. It is thus evident that hydrogen is more effective than other antiaging drugs. In addition, *age-1* mutants were more resistant to copper than wild-type worms and revealed an increased capacity for antioxidant enzyme activity and expression during copper treatment[27]. *Ins-18*, with a C peptide, antagonizes *daf-2*, and a high gene dosage of *ins-18* induces dauer arrest in wild-type animals at 26°C and enhances dauer arrest in *daf-2*(e1365) at 20°C[28]. In this paper, the expression of *age-1* and *let-363* genes was downregulated, and the expression of the *ins-18* gene was upregulated at the same age after hydrogen treatment, providing further evidence to support the beneficial effect of hydrogen on worms. The abovementioned results further indicated that hydrogen might make nematodes younger and more vigorous and allow them to resist the effects of aging.

SOD is an antioxidant metalloproteinase that exists in organisms. It can catalyze the disproportionation of superoxide anion radicals to produce oxygen and hydrogen peroxide. SOD plays an important role in the balance between oxidation and antioxidation and is closely related to the occurrence and development of many diseases[29]. *Sod-5* is necessary for copper detoxification, and other genes also play a role in copper detoxification[27]. The mRNA expression levels of *sod-1*, *sod-2*, *sod-3* and *sod-5* were upregulated under 0.25 mM phoxim treatment, especially *sod-3* and *sod-5*, which increased >10- and 70-fold, respectively[30]. In addition, studies have also shown that the higher the survival rate of *C*. *elegans* under stress conditions, the better the life expectancy is, indicating that the prolongation of life has a strong relationship with an increased survival rate under stress[31]. For the above reasons, different concentrations of PQ were used to investigate and estimate the protective mechanism of hydrogen in this paper. After hydrogen treatment in PQ-treated worms, the ROS level of N2 and *sod-5* mutant strains decreased significantly, and longevity was also extended, although the ROS level of the *sod-5* mutant strains was still much higher than that of the N2 strains, and longevity was shorter. As a result of a SOD defect, *C*. *elegans* cannot rapidly eliminate ROS, leading to excessive ROS accumulation *in vivo*. In addition, PQ is known to cause changes in worm physiology due to oxidative damage, interfere with electron transfer and catalyze the production of ROS[32,33]. When *C*. *elegans* were treated with 0.5 mM PQ for 1, 3 and 5 days, our results showed that the body length of N2 and *sod-5* strains in the PQ and hydrogen groups was significantly smaller than that of the control group, but compared with that of the PQ group, the body length of *C*. *elegans* in the hydrogen group was partially recovered. The adult rate and spawning number were much lower in the 0.5 mM PQ group than in the control group but increased significantly after hydrogen treatment. However, under the 5 mM PQ condition, the lifespan of the N2 strain could be extended, while the lifespan of the *sod-5* strains was not obviously altered after hydrogen treatment. It is important that the ROS levels decreased significantly and the longevity was extended in the hydrogen group after 0.5 mM PQ treatment. It is thus clear that hydrogen can significantly resist 0.5 mM PQ, which usually leads to damage to *C*. *elegans*, including aging, stem length, adult rate and spawning number; this result further proves that oxidative stress is one of the causes of senescence[5,34]. However, Seung-Jae Lee *et al*. reported that low paraquat levels (0.125 mM, 0.25 mM, 0.5 mM, and 1 mM) increased life span significantly, whereas, as expected, higher concentrations of paraquat (4, 16, and 64 mM) decreased life span in a dose-dependent manner[35]. In this paper, the PQ concentration chosen was based on a previous study[14], where 30% worm mortality and ROS enhancement were observed at a PQ concentration of 0.5 mM compared to the control group. Therefore, further research is needed in the future.

Overall, our current work showed that the protective function of hydrogen is closely related to ROS[36], indicating that hydrogen as a supplemental exogenous antioxidant can neutralize the active oxygen forms produced in organisms and reduce the damage due to ROS. Therefore, hydrogen treatment could be used as a valuable potential approach to increasing longevity and health through reducing ROS.

## Conclusion

Taken together, our data showed that hydrogen is a good antioxidant that can significantly reduce the body’s ROS levels and extend the lifespan of *C*. *elegans*.

## Supporting information

S1 Fig Installation diagram of hydrogen treatment. (TIF)

S2 Fig Schematic diagram of hydrogen treatment and related index detection. Body length assay: 1 d, 3 d and 5 d; Reproduction assay: 2 d; ROS assay: 7 d and 14 d; Lifespan assay: 10 d; Gene expression assay: 15 d.
(TIF)

S3 Fig N2 and *sod-5* nematodes spawning with or without hydrogen treatment per day. Data are shown as the mean ± SD of three independent experiments. * *p<0*.*05*; ** *p<0*.*01*.
(TIF)

S4 Fig N2 nematodes spawning with or without PQ treatment after hydrogen per day. Data are shown as the mean ± SD of three independent experiments. * *p<0*.*05*; ** *p<0*.*01*.
(TIF)

S5 Fig *Sod-5* nematodes spawning with or without PQ treatment after hydrogen per day. Data are shown as the mean ± SD of three independent experiments. * *p<0*.*05*; ** *p<0*.*01*.
(TIF)

S1 Raw images (ZIP)
