# Correction: Hydrogen extends *Caenorhabditis elegans* longevity by reducing reactive oxygen species

**DOI:** 10.1371/journal.pone.0234274

**Published:** 2020-05-29

**Authors:** The PLOS ONE Staff

The fourth author, Wenjing Gong, is incorrectly noted as one of the corresponding authors. The correct corresponding author is Zhihui Li. Zhihui Li's email address is correctly listed as: l3521025015@163.com. The publisher apologizes for the error.

The ORCID iD is missing for the sixth author. Author Chenggang Zhang’s ORCID iD is: 0000-0002-4521-3304 (https://orcid.org/0000-0002-4521-3304).
