# Supplementary figures and images for: Hydrogen extends Caenorhabditis elegans longevity by reducing reactive oxygen species
Source: PLoS One. 2020 Apr 22;15(4):e0231972. doi: 10.1371/journal.pone.0231972 (PMC7176462; doi:10.1371/journal.pone.0231972)

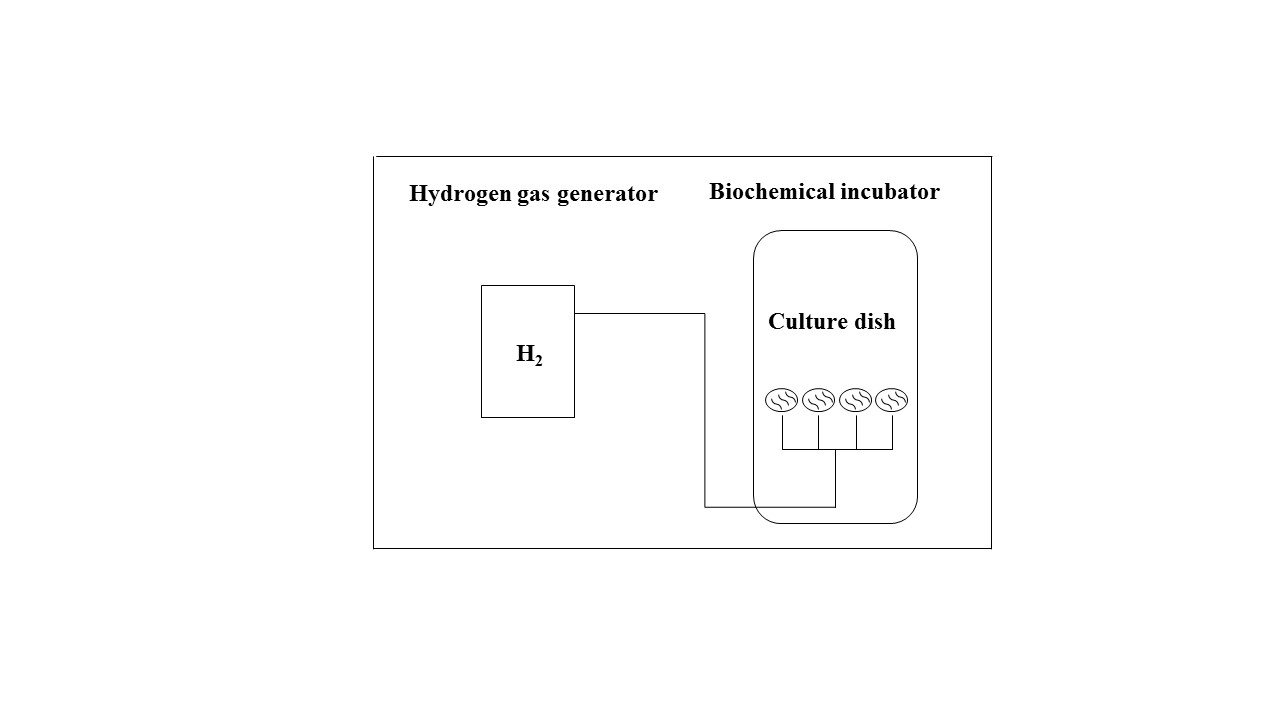

Supplement: S1 Fig — (TIF) [file pone.0231972.s001.tif]

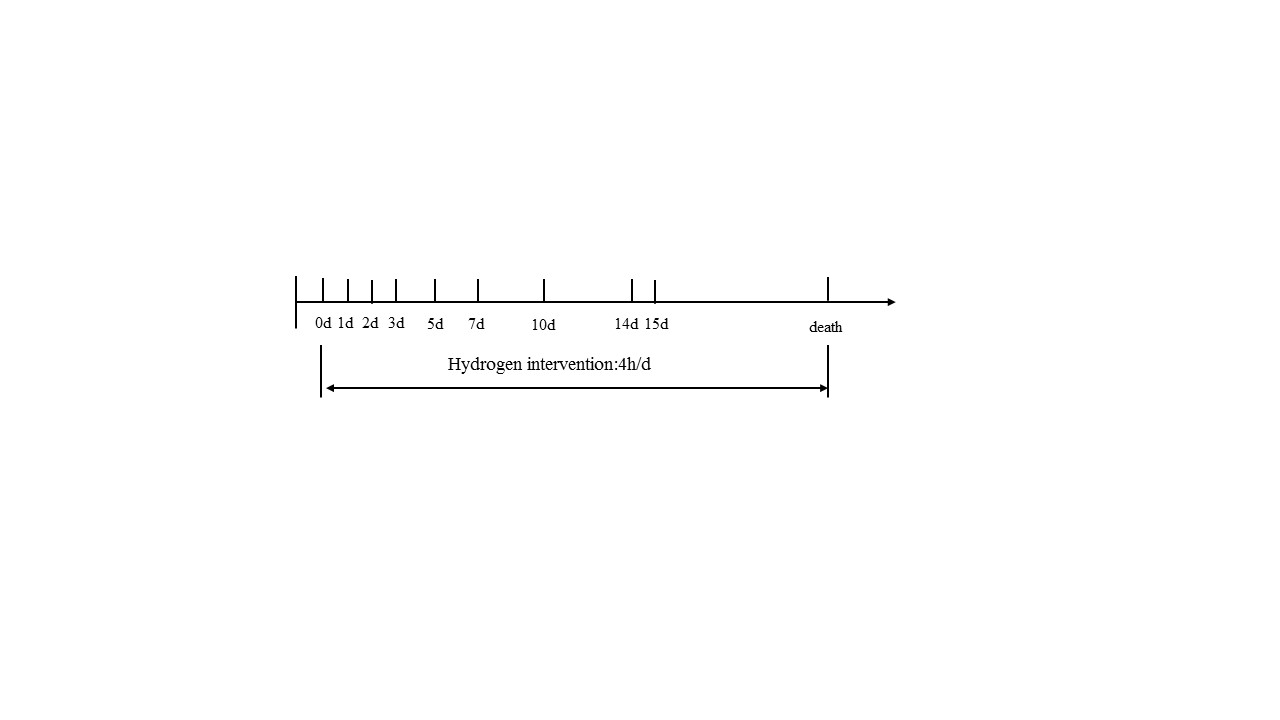

Supplement: S2 Fig — Body length assay: 1 d, 3 d and 5 d; Reproduction assay: 2 d; ROS assay: 7 d and 14 d; Lifespan assay: 10 d; Gene expression assay: 15 d. (TIF) [file pone.0231972.s002.tif]

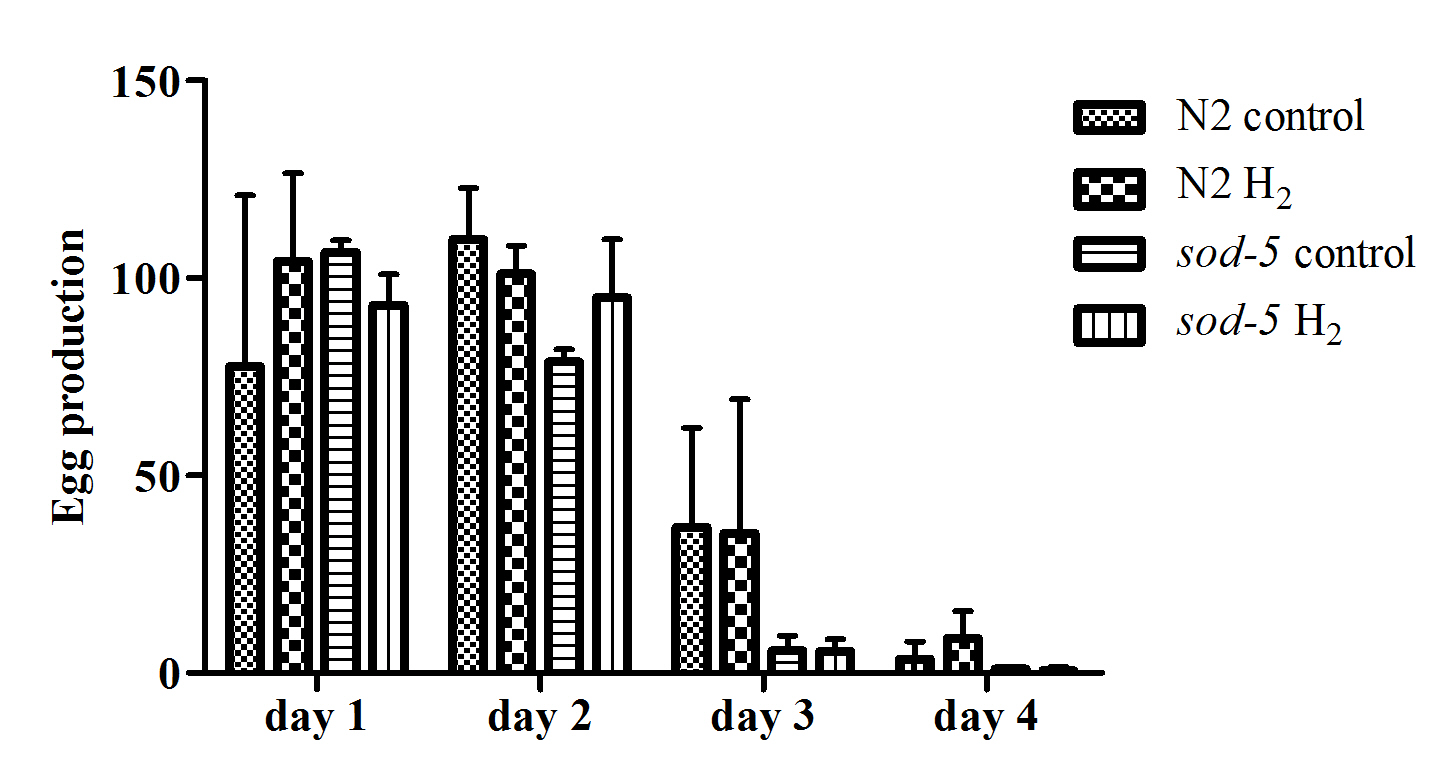

Supplement: S3 Fig — Data are shown as the mean ± SD of three independent experiments. * p<0.05; ** p<0.01. (TIF) [file pone.0231972.s003.tif]

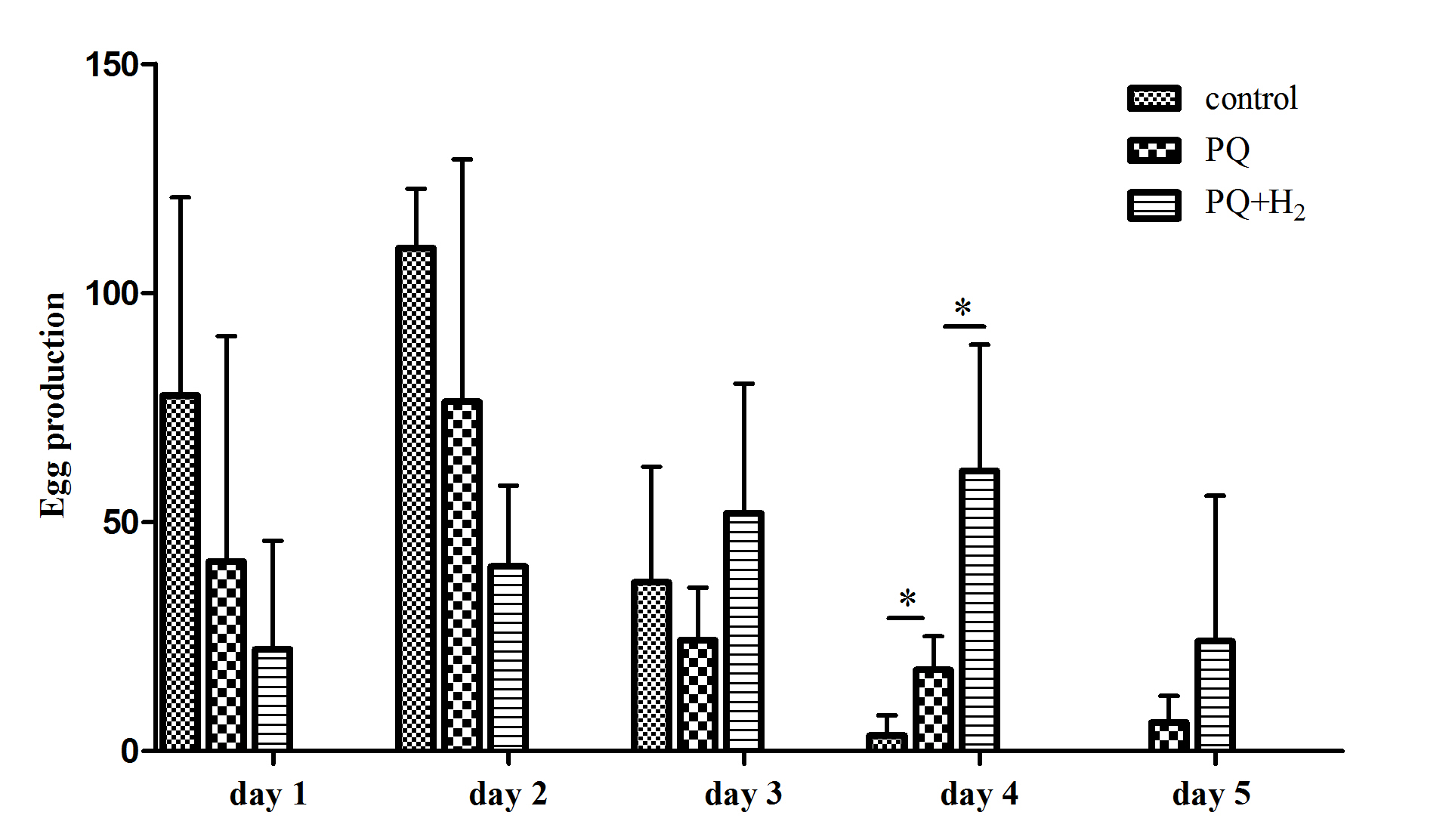

Supplement: S4 Fig — Data are shown as the mean ± SD of three independent experiments. * p<0.05; ** p<0.01. (TIF) [file pone.0231972.s004.tif]

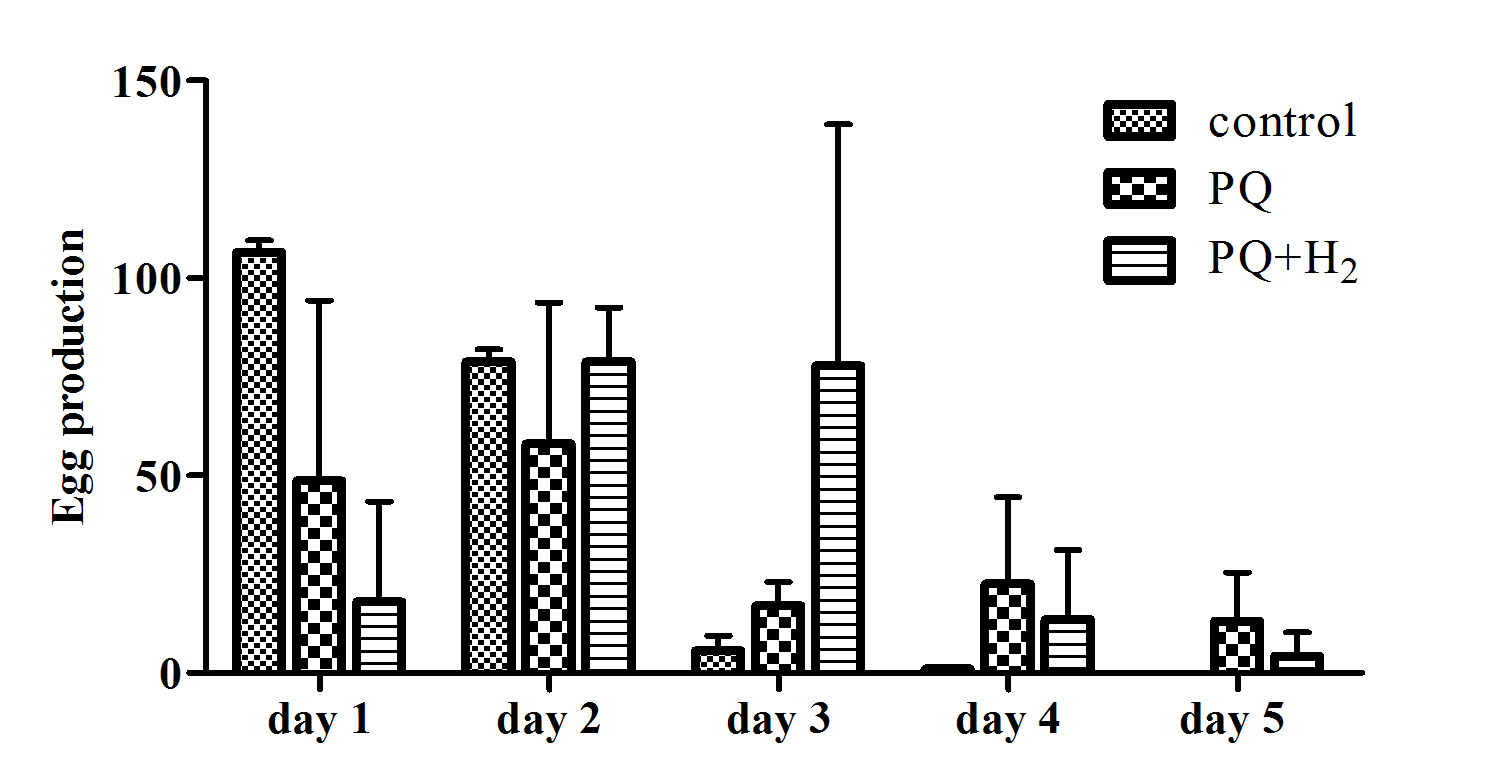

Supplement: S5 Fig — Data are shown as the mean ± SD of three independent experiments. * p<0.05; ** p<0.01. (TIF) [file pone.0231972.s005.tif]
