# Supplementary material for: Hydrogen extends Caenorhabditis elegans longevity by reducing reactive oxygen species
Source: PLoS One. 2020 Apr 22;15(4):e0231972. doi: 10.1371/journal.pone.0231972 (PMC7176462; doi:10.1371/journal.pone.0231972)

Fig 4A

N2-0.5mM PQ-control-1d

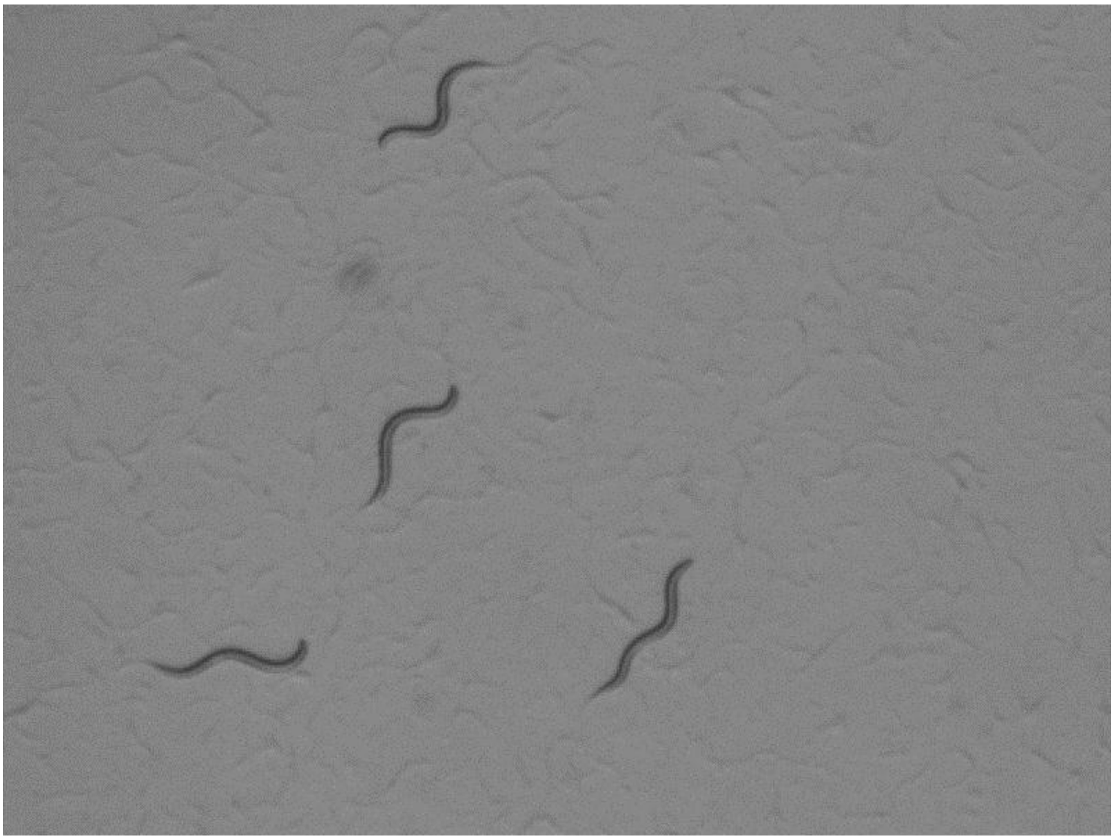

N2-0.5mM PQ-control-3d

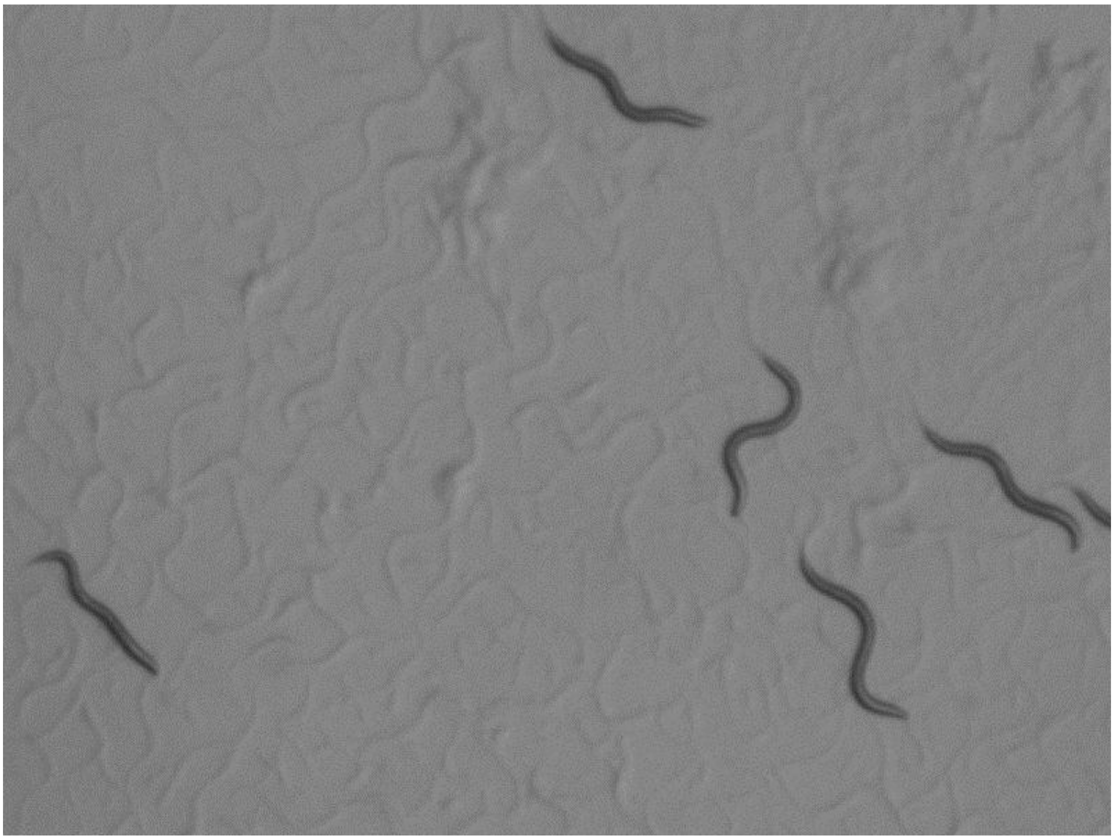

N2-0.5mM PQ-control-5d

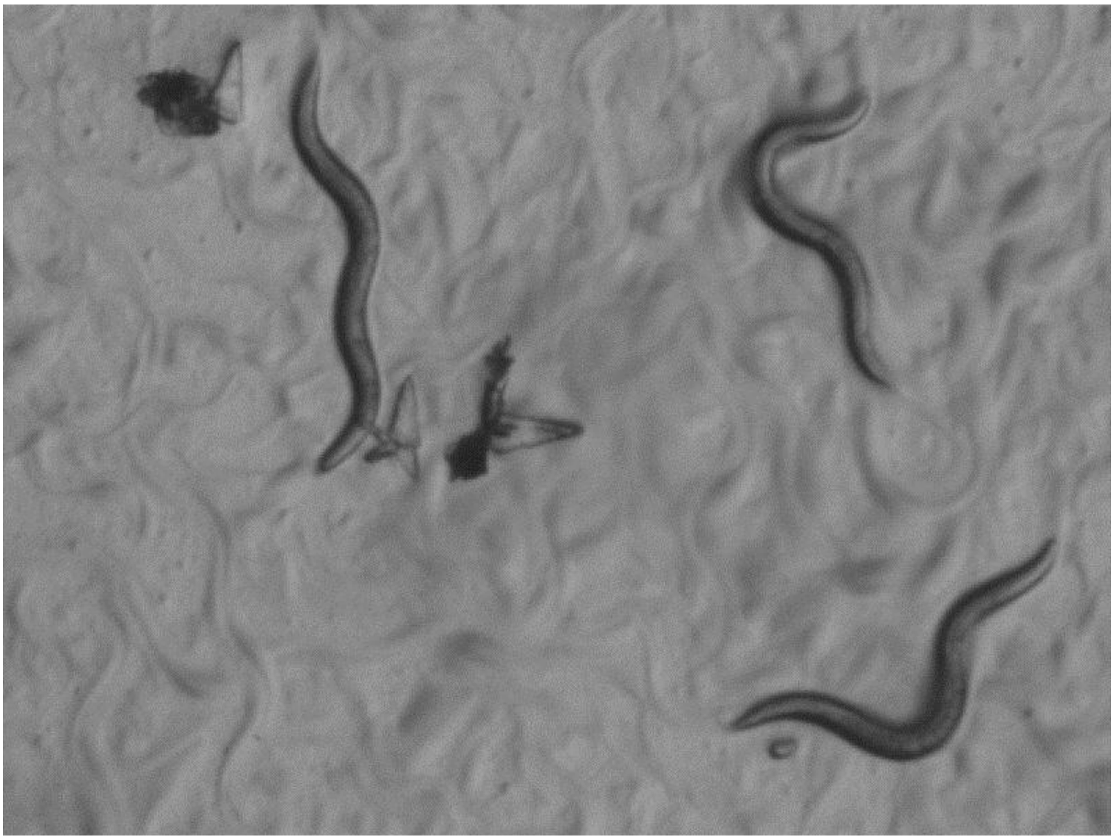

N2-0.5mM PQ-H_2-1d

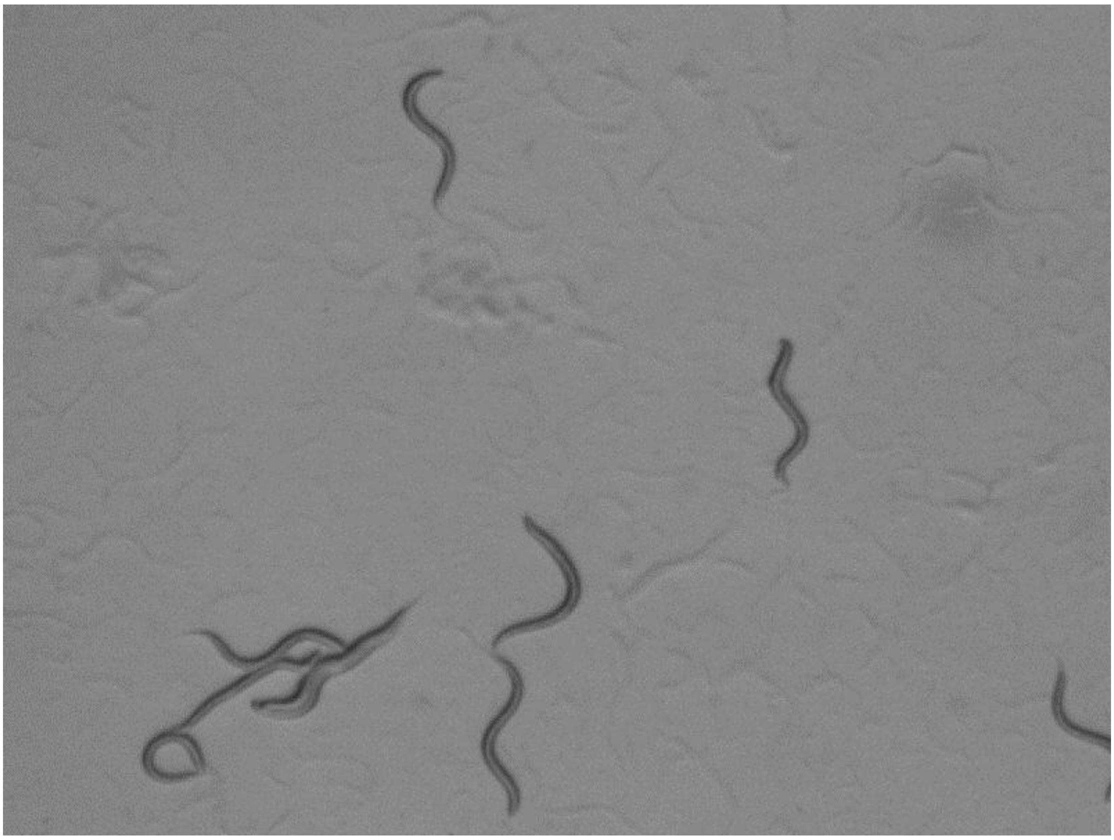

N2-0.5mM PQ-H_2-3d

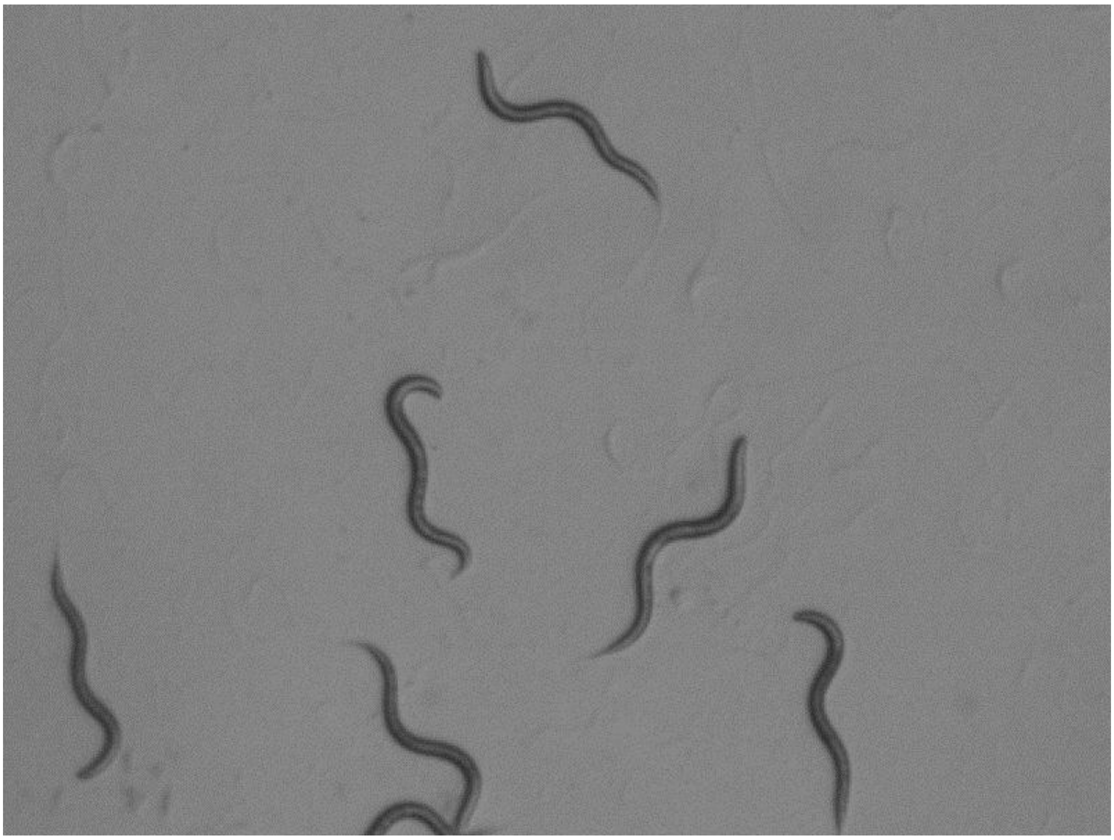

N2-0.5mM PQ-H_2-5d

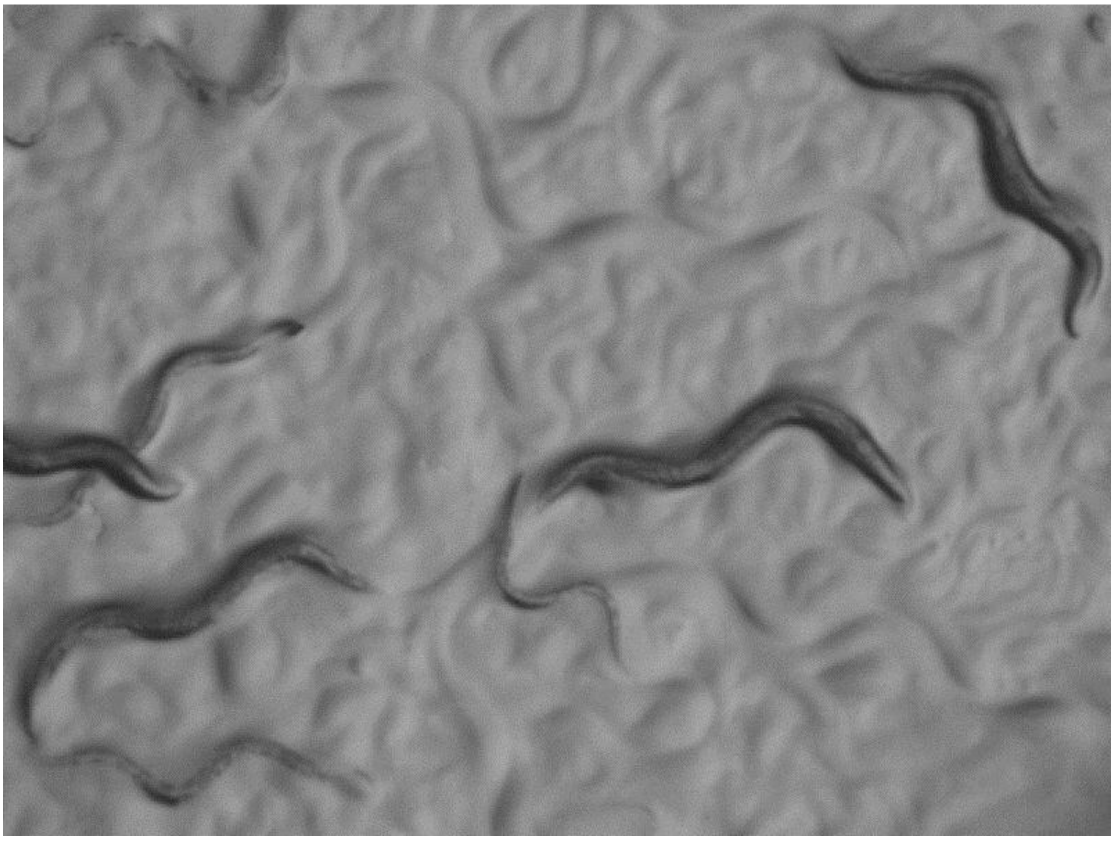

N2-control-1d

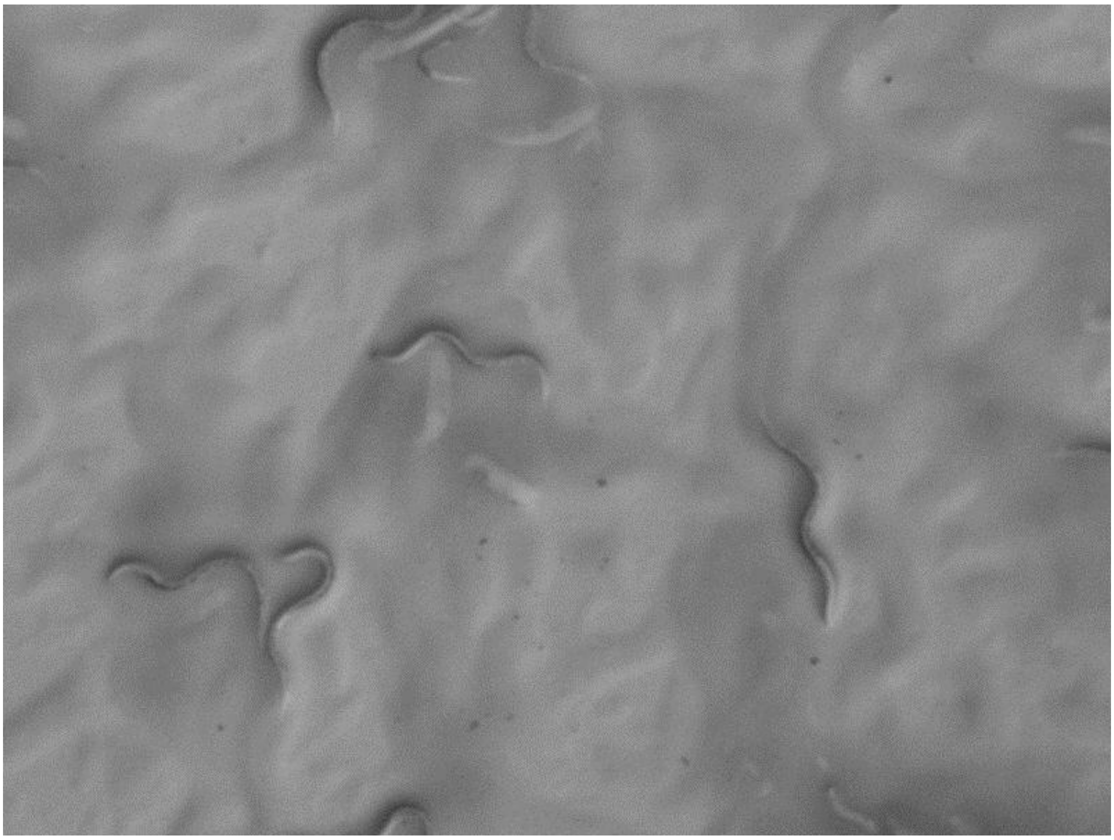

N2-control-3d

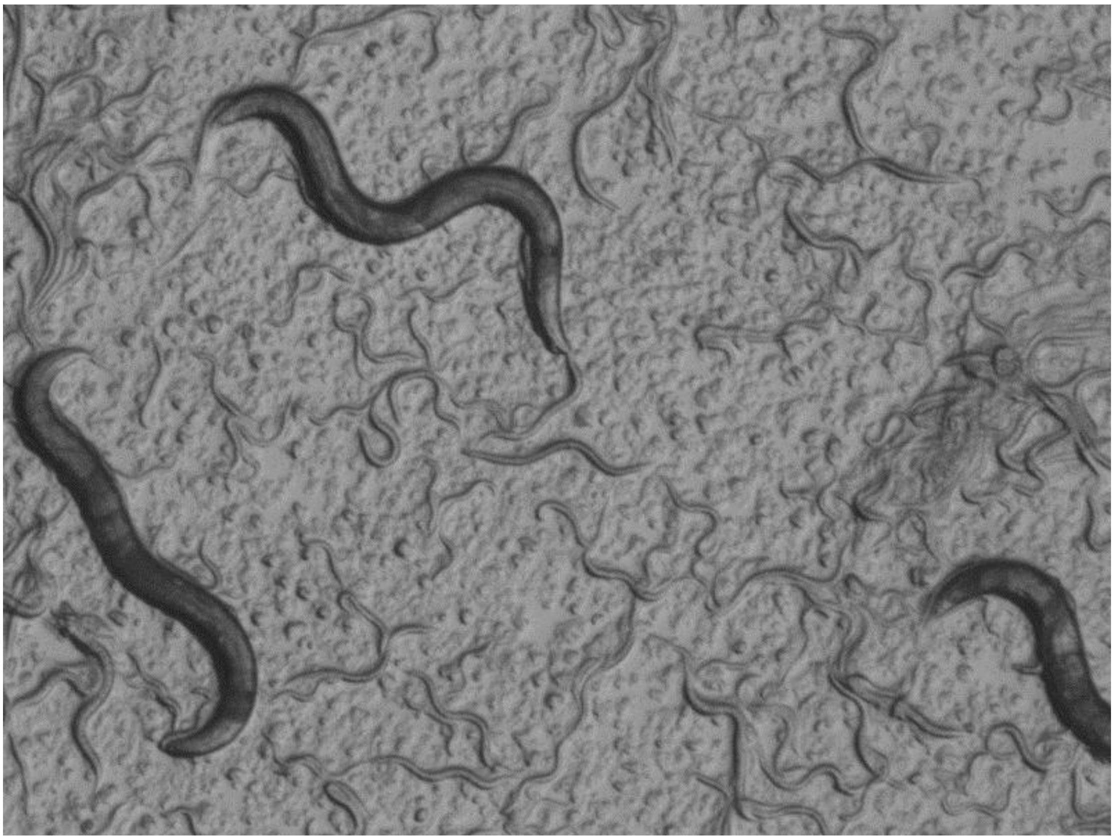

N2-control-5d

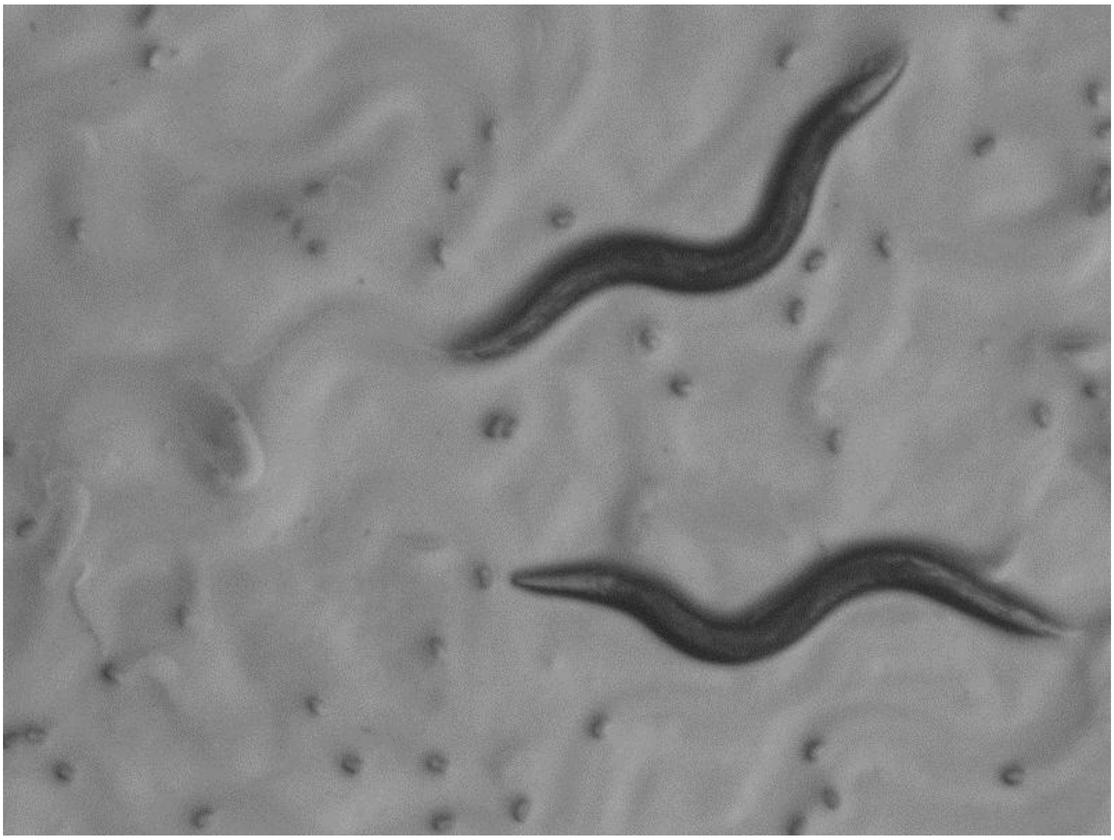

Fig 4C

Sod-5-0.5mM PQ-control-1d

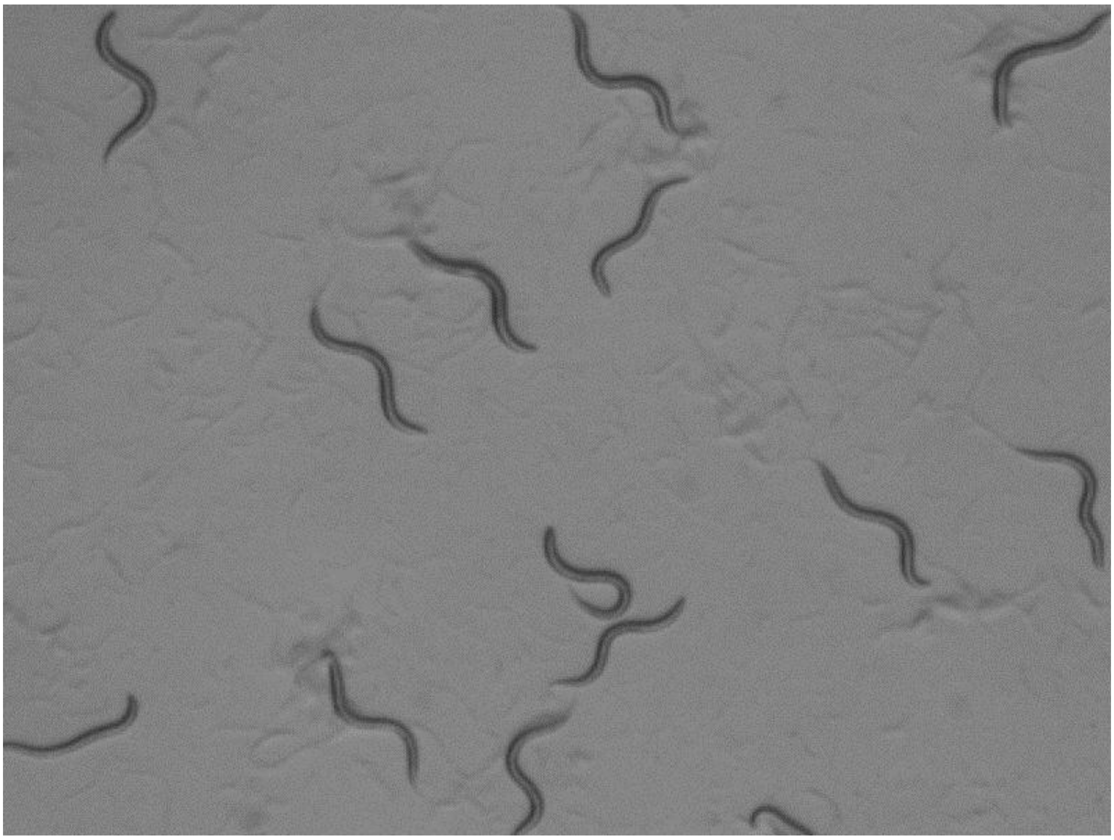

Sod-5-0.5mM PQ-control-3d

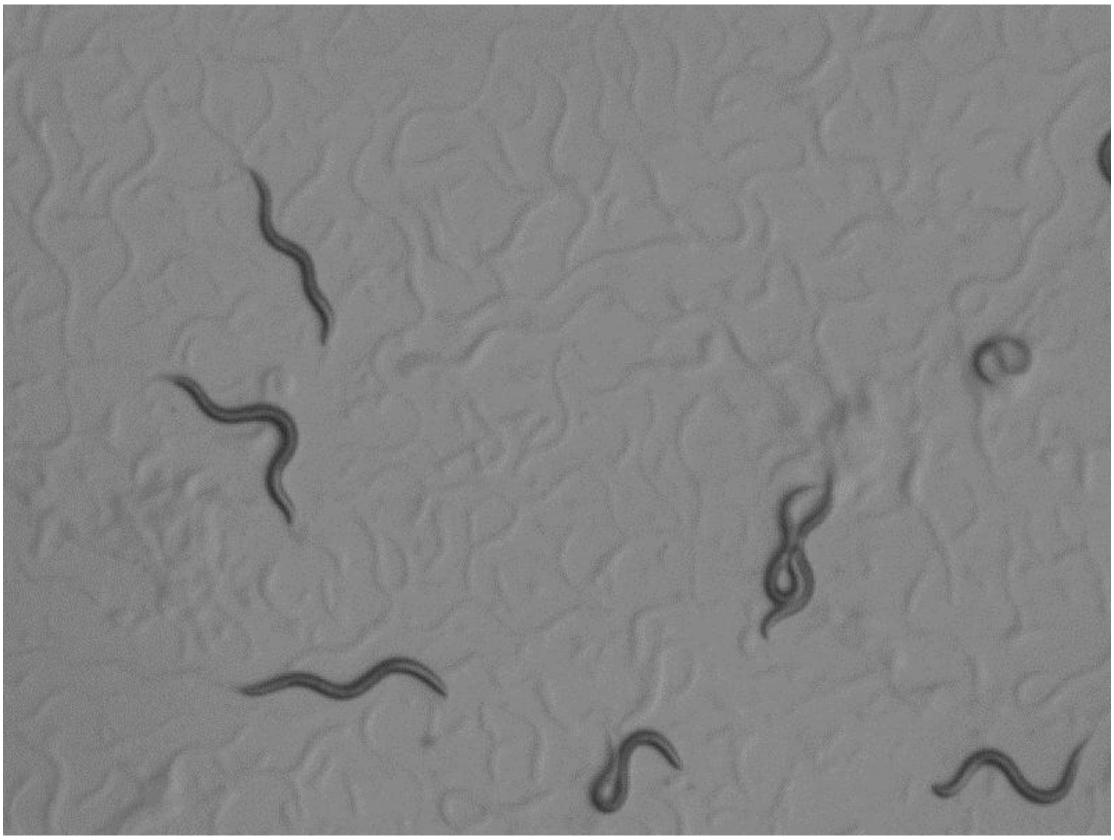

Sod-5-0.5mM PQ-control-5d

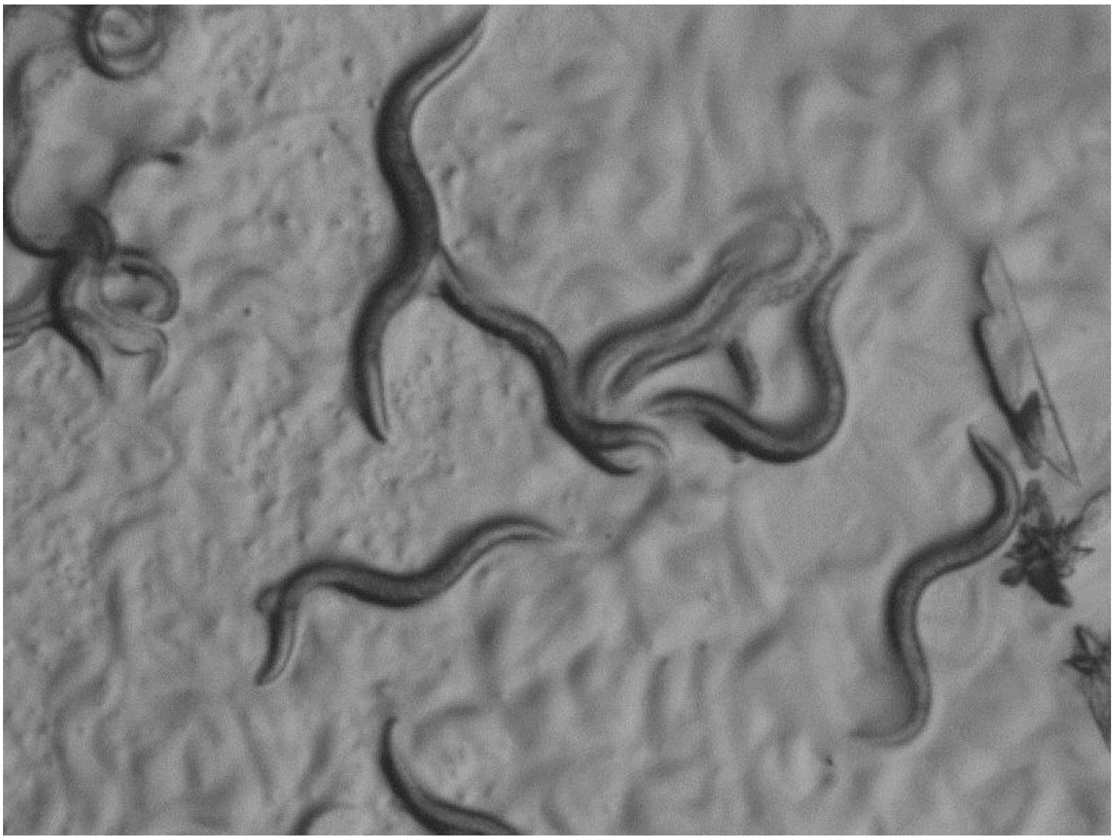

Sod-5-0.5mM PQ-H_2-1d

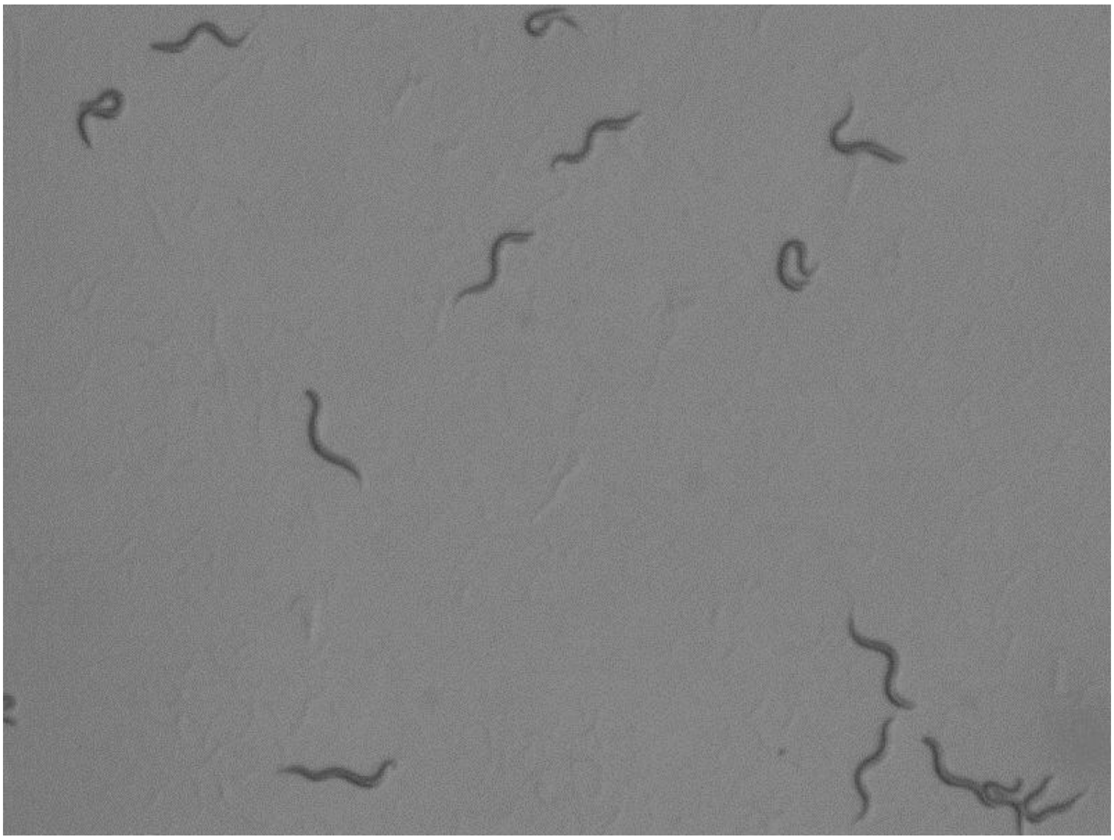

Sod-5-0.5mM PQ-H_2-3d

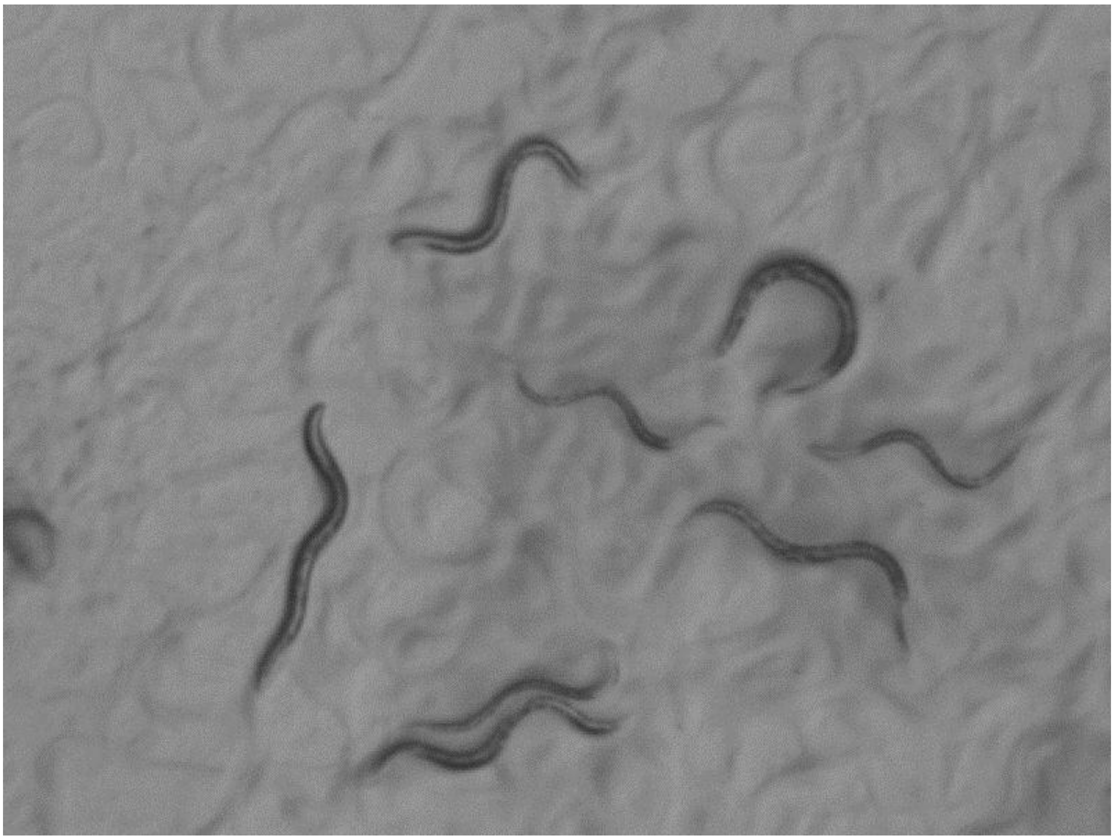

Sod-5-0.5mM PQ-H_2-5d

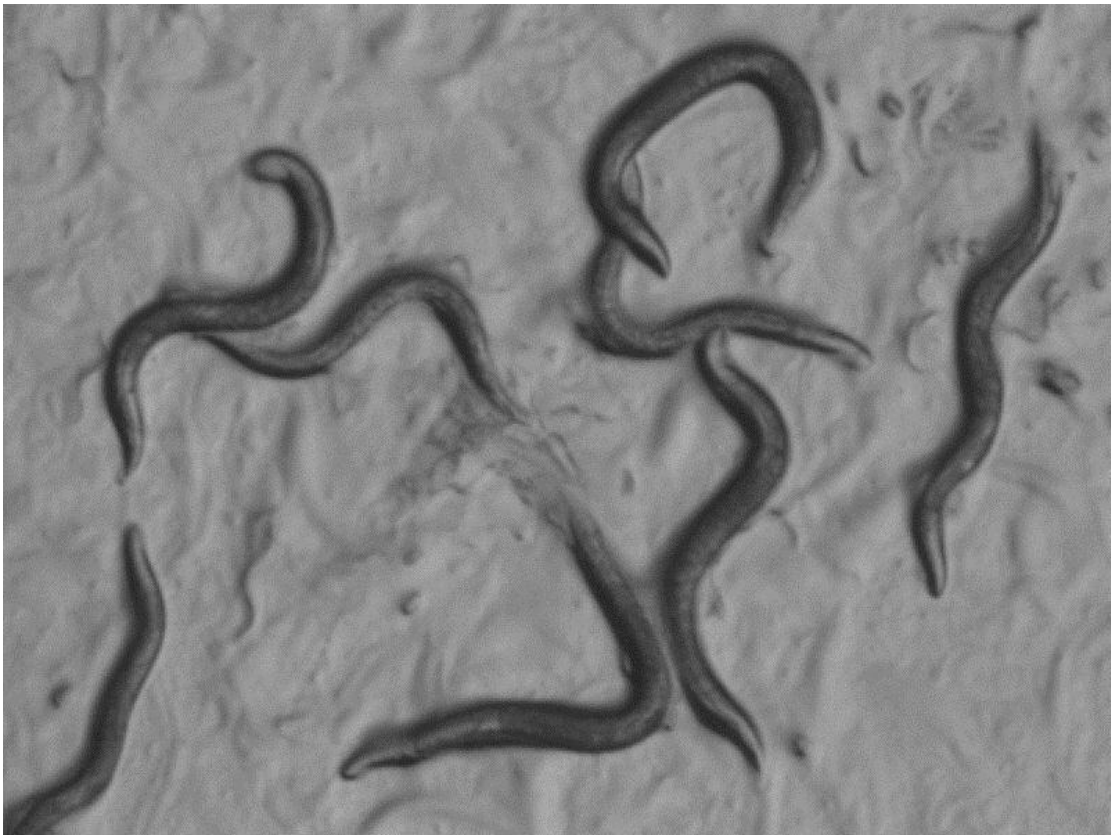

Sod-5-control-1d

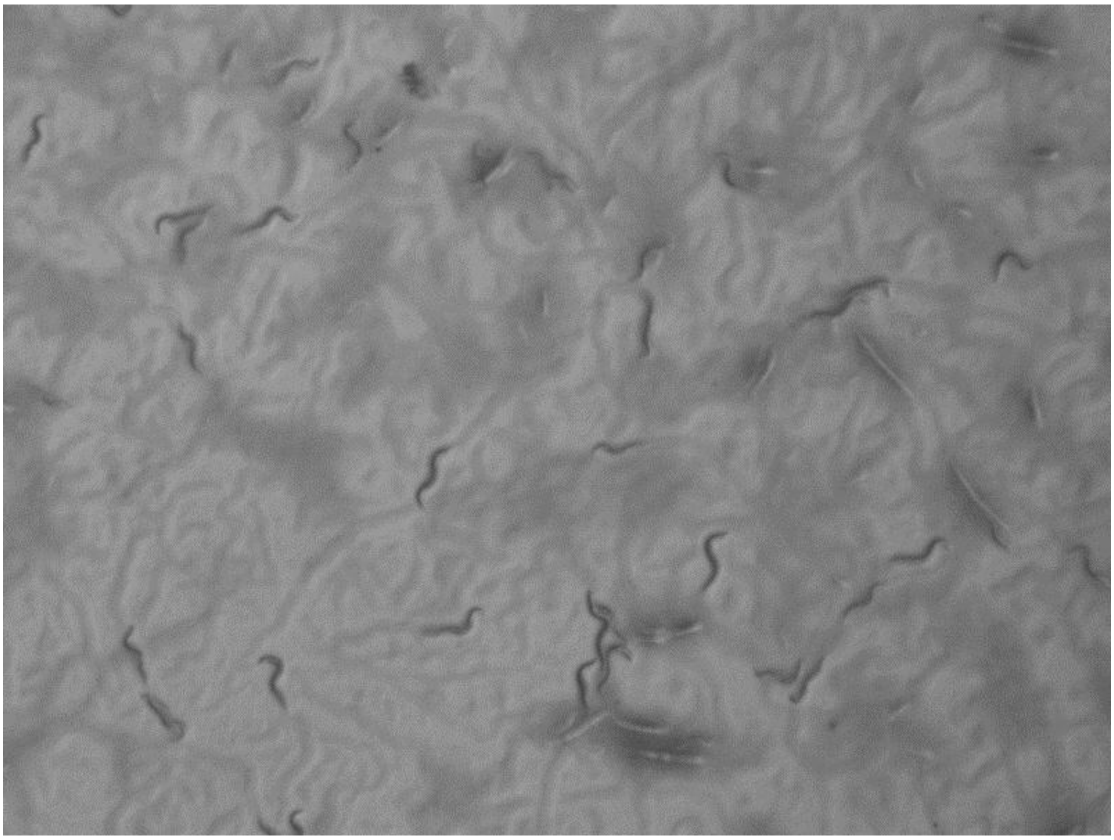

Sod-5-control-3d

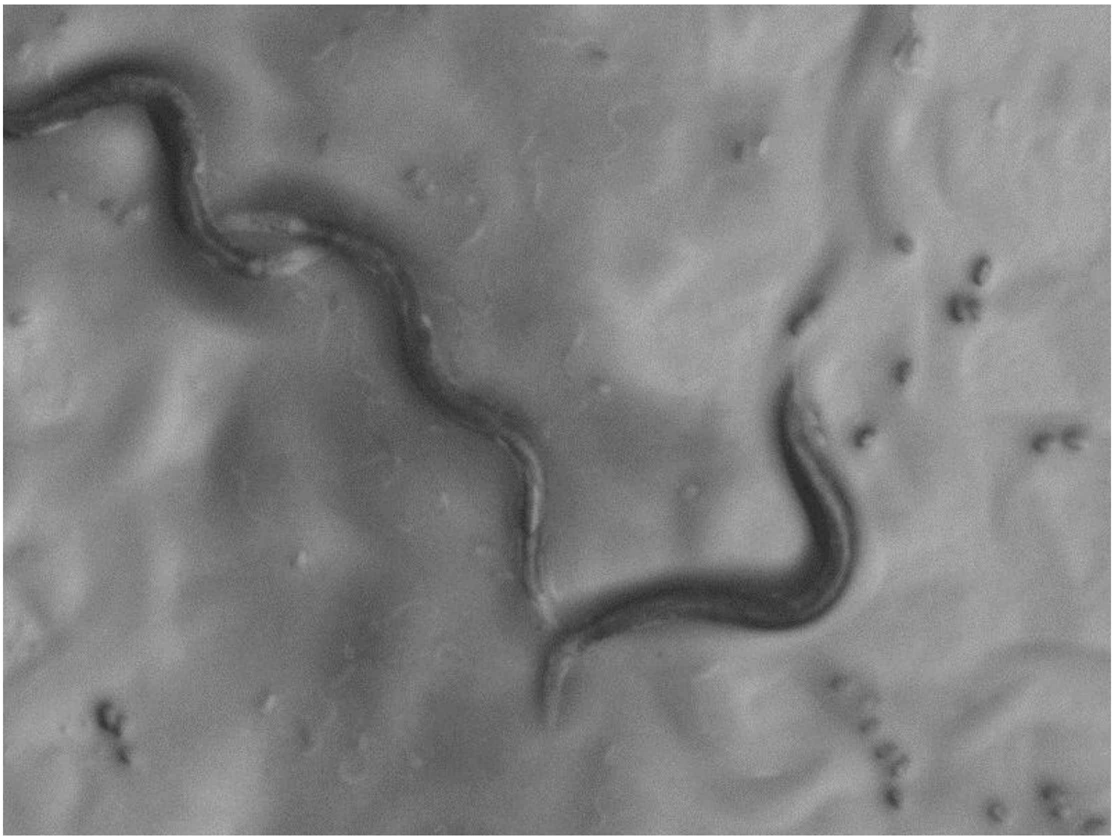

Sod-5-control-5d

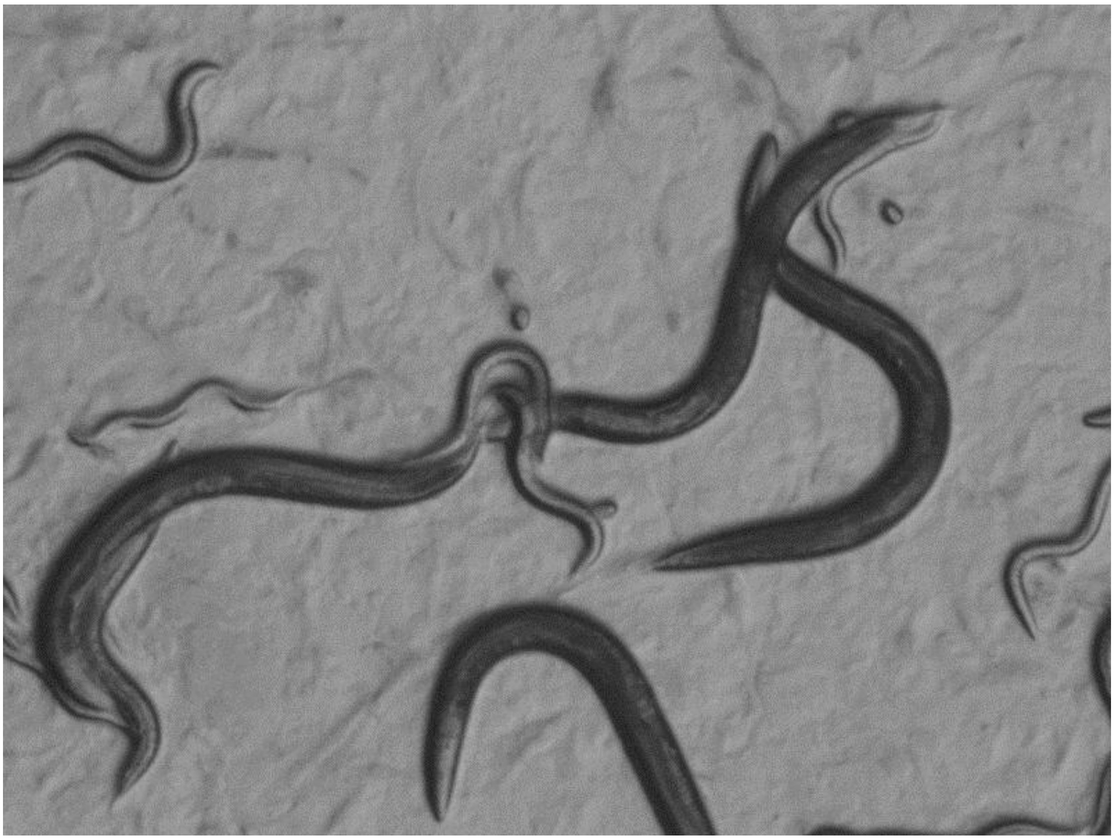

Fig 2B

N2-control-7d

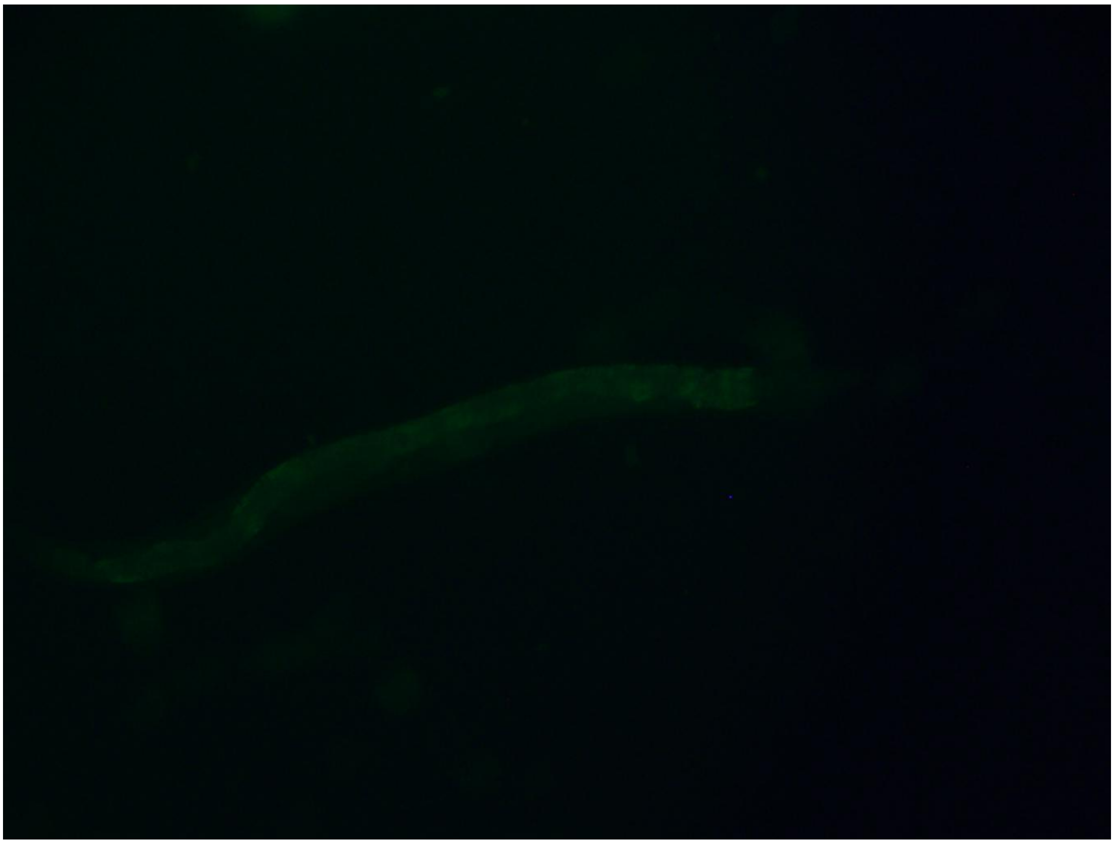

N2-control-14d

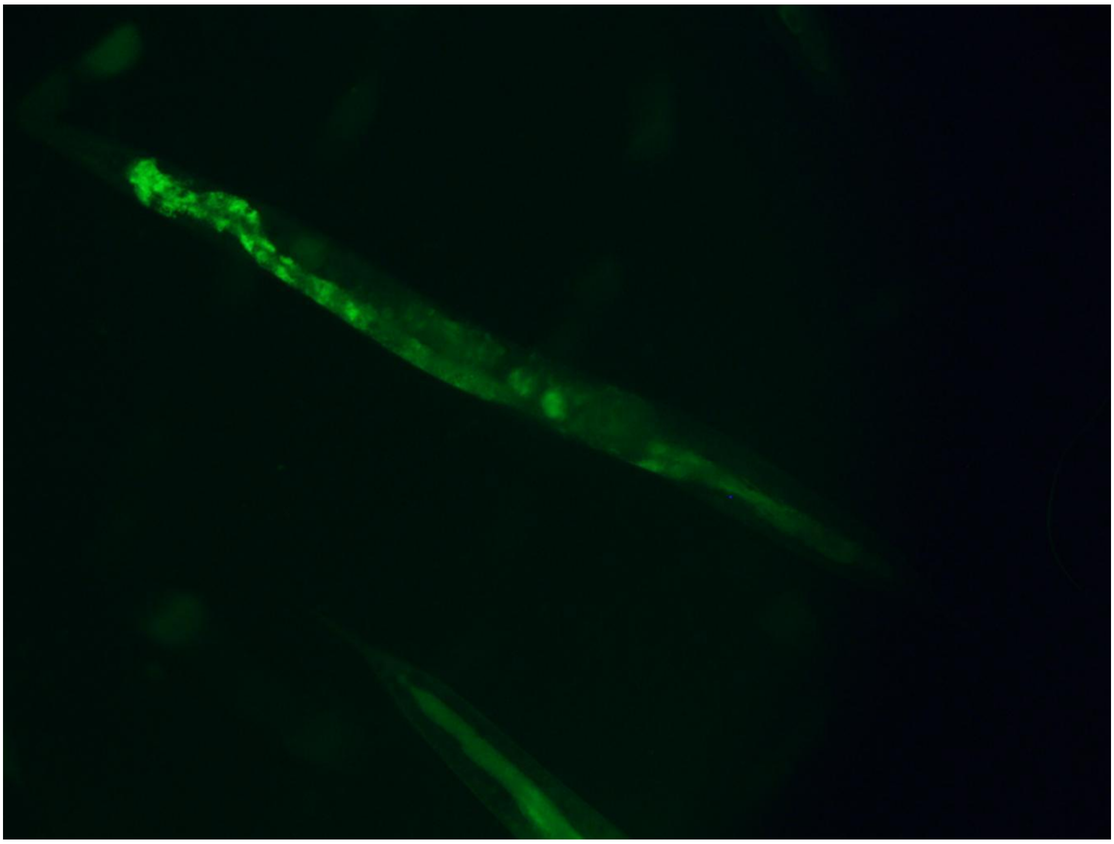

Sod-5-control-7d

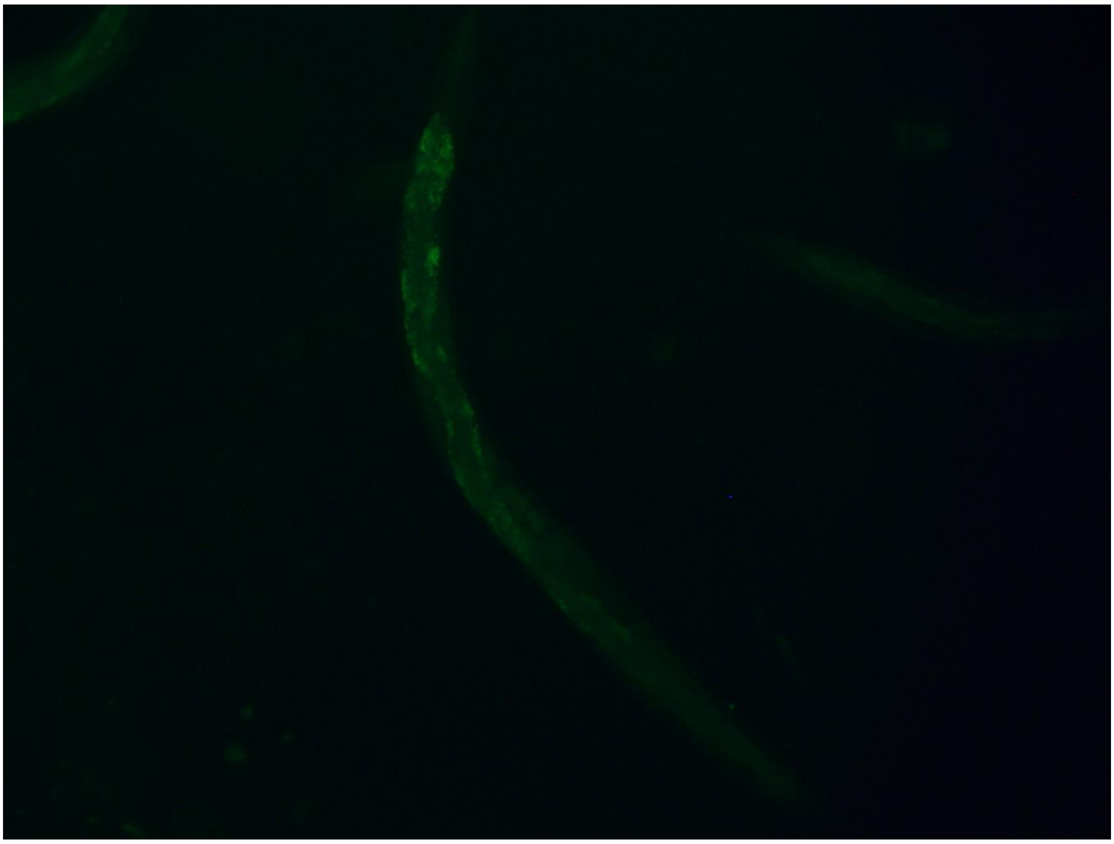

Sod-5-control-14d

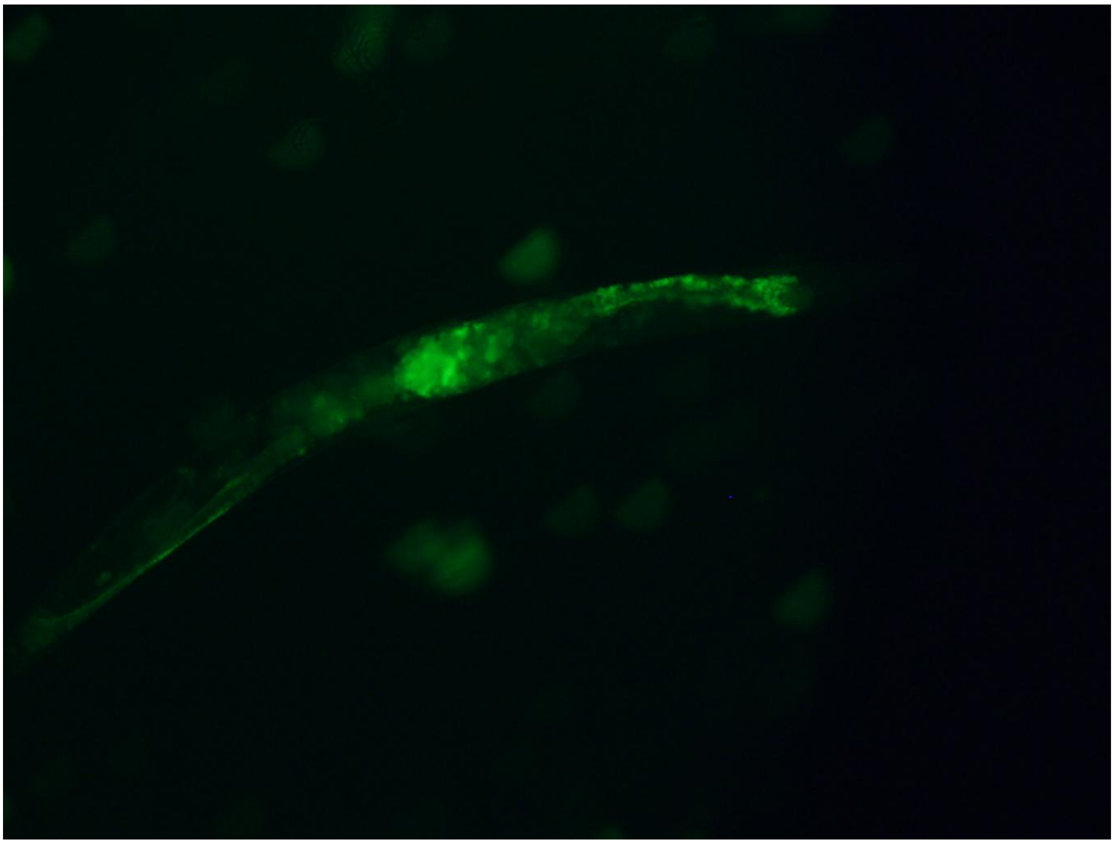

Fig 2D

N2-control-14d

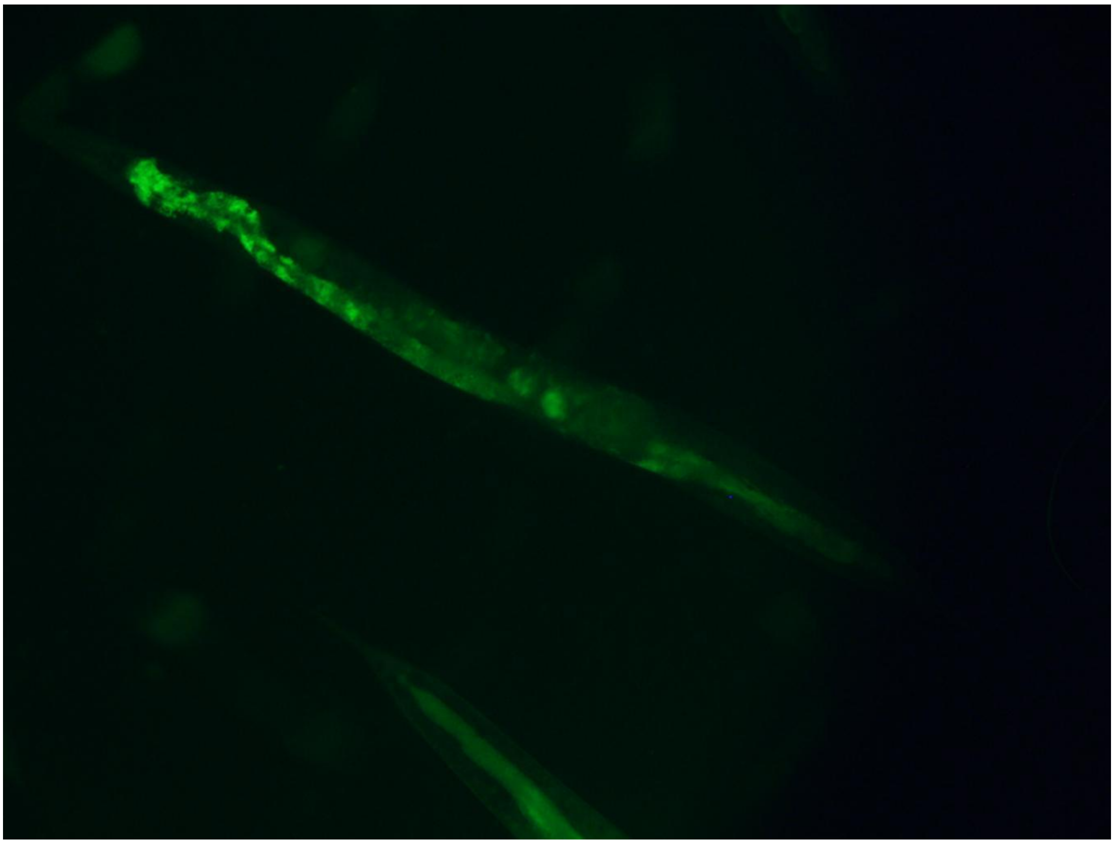

N2-H_2-14d

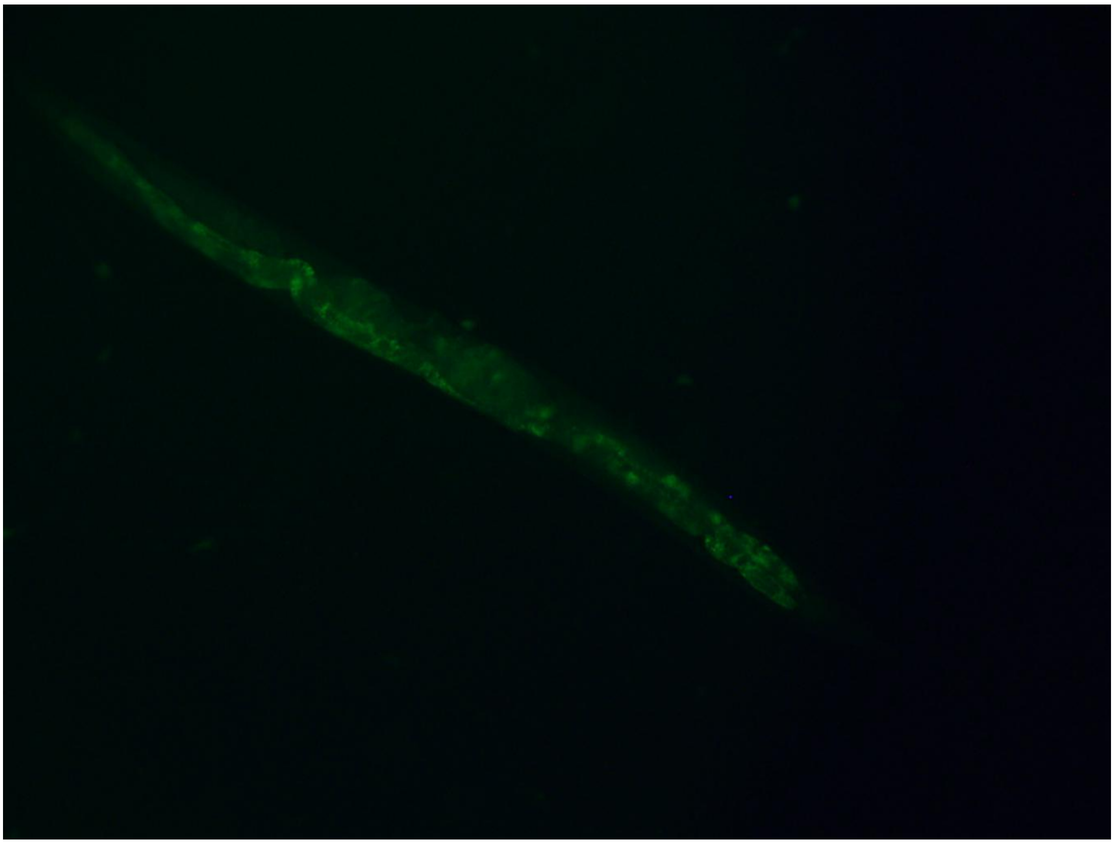

Sod-5-control-14d

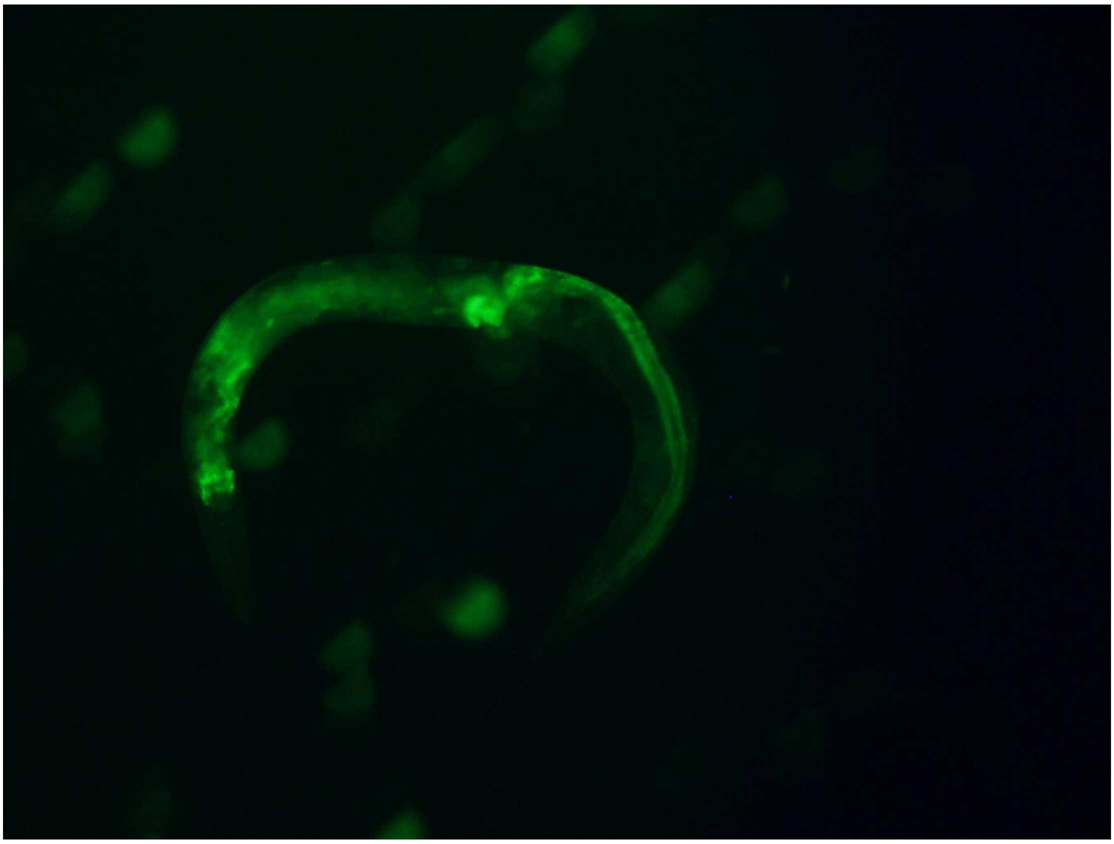

Sod-5-H_2-14d

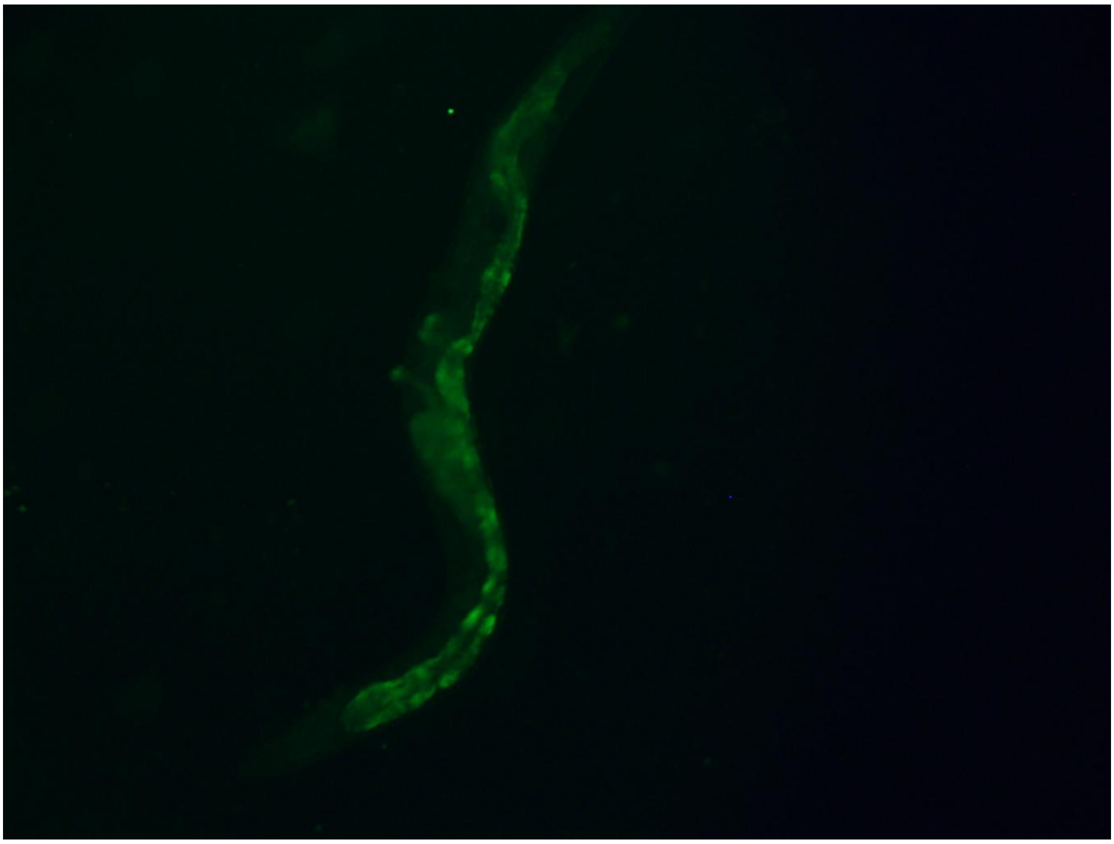

Fig 3

age-1

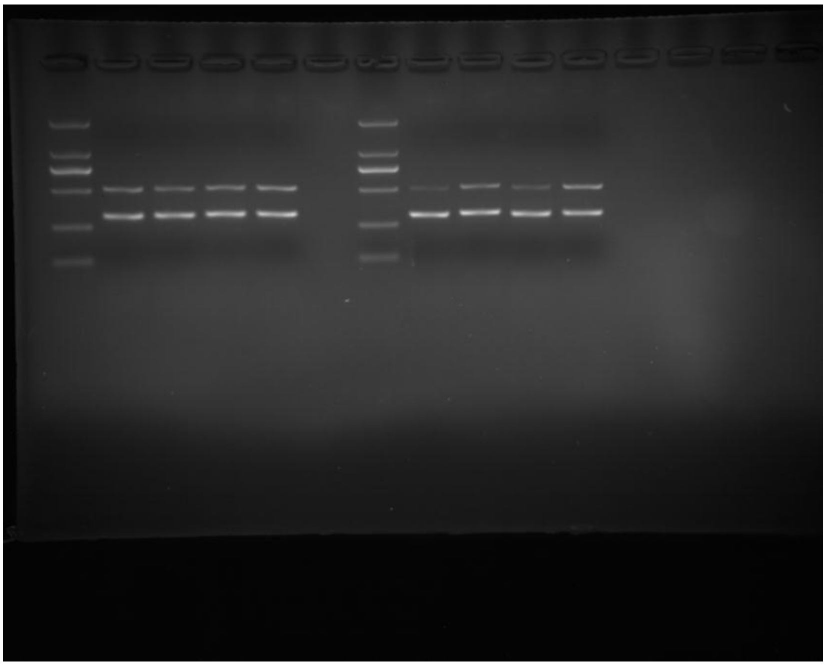

let-363

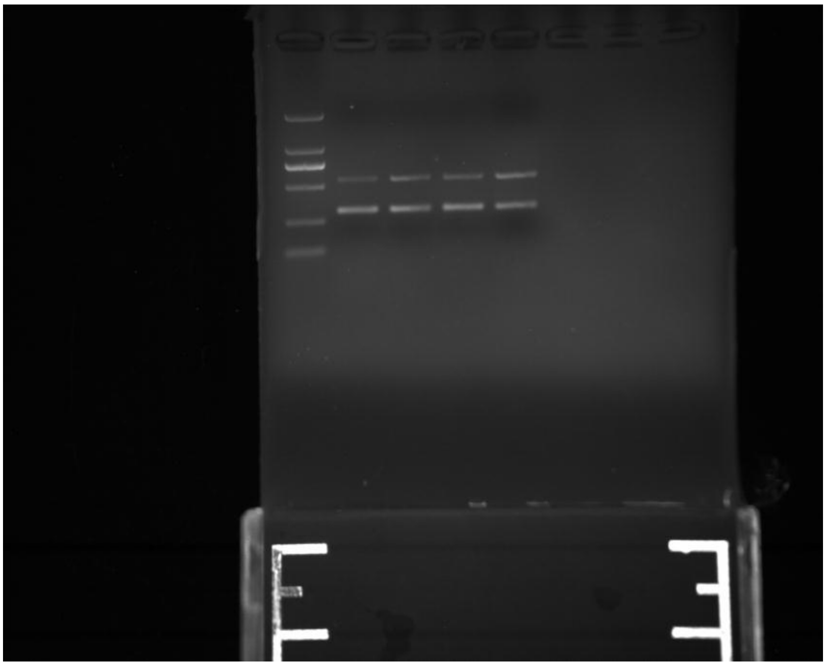

ins-18

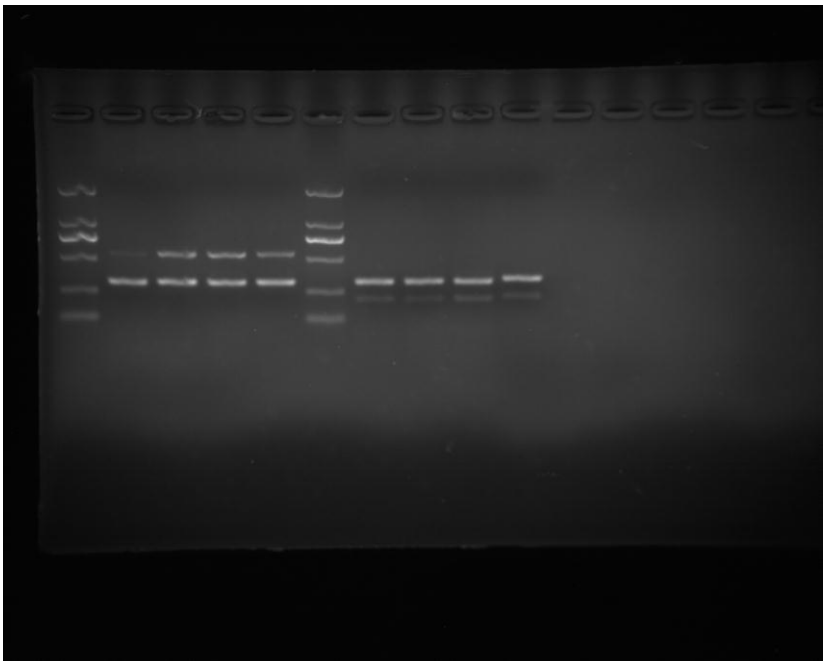

Fig 5A

N2-0.5mM PQ-H_2

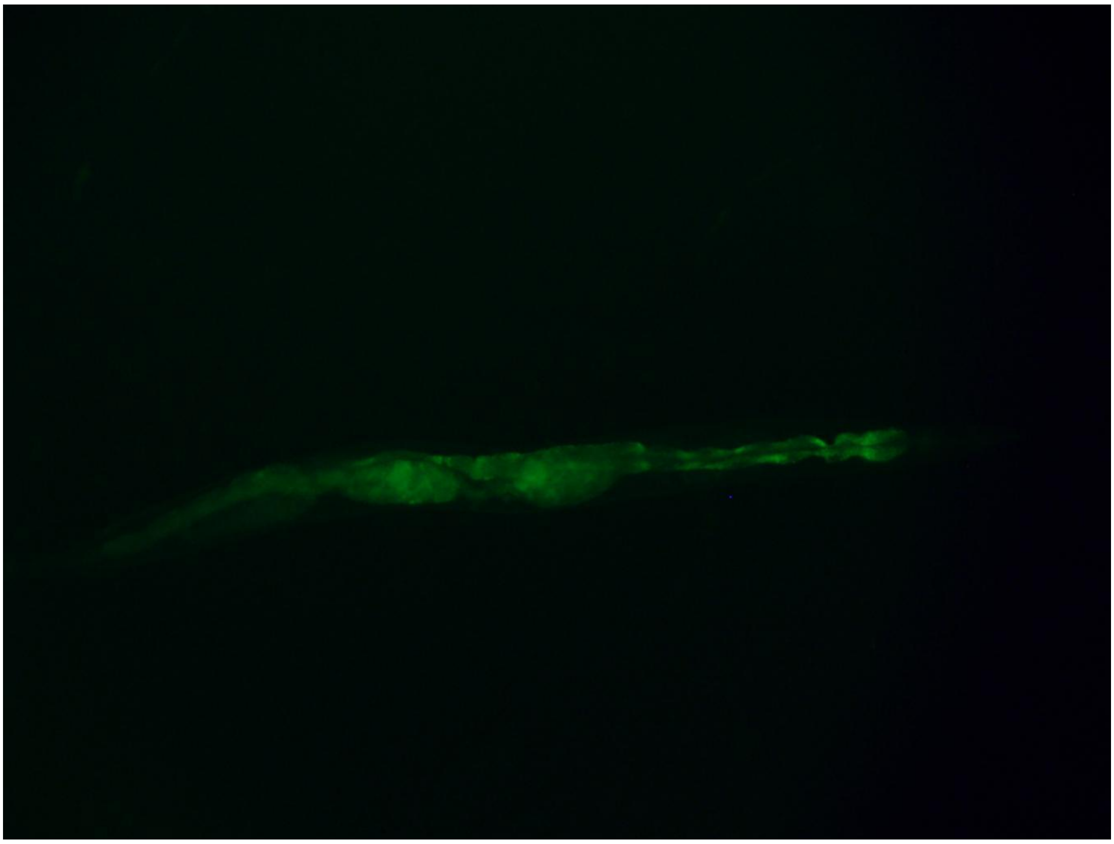

N2-0.5mM PQ-control

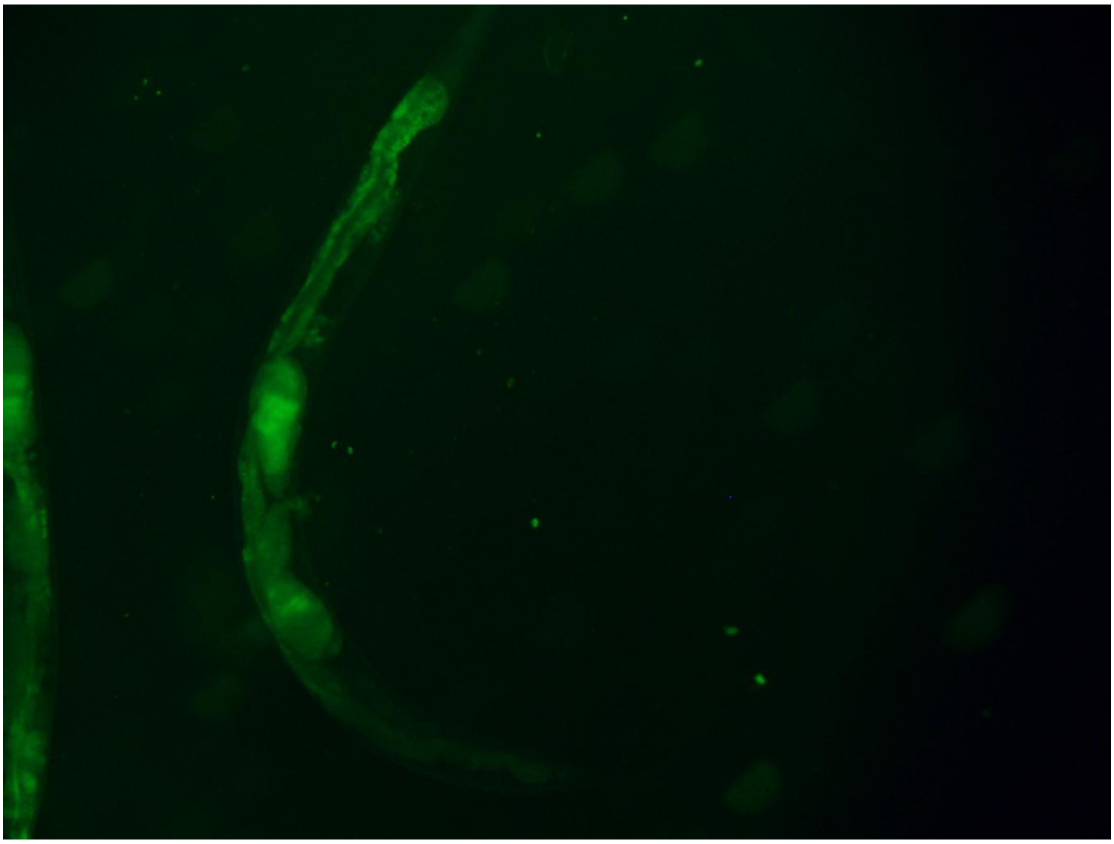

Fig 5C

Sod-5-0.5mM PQ-control

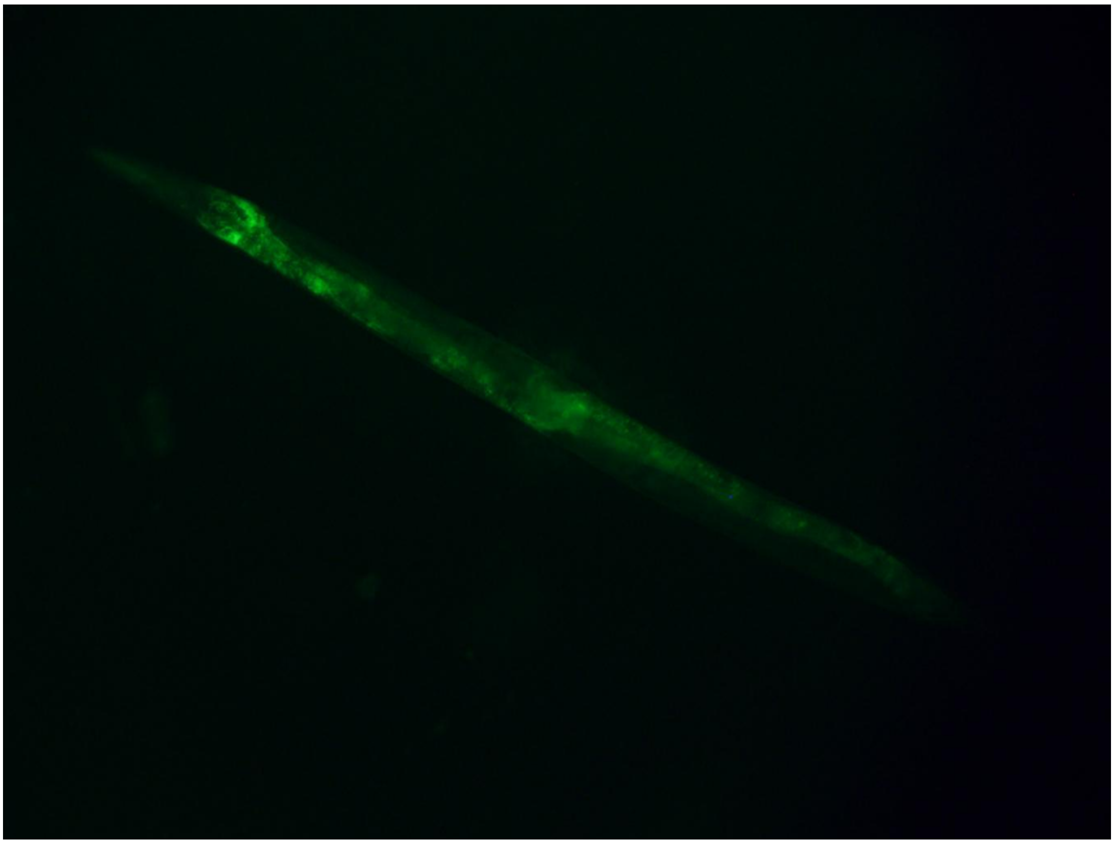

Sod-5-0.5mM PQ-H_2

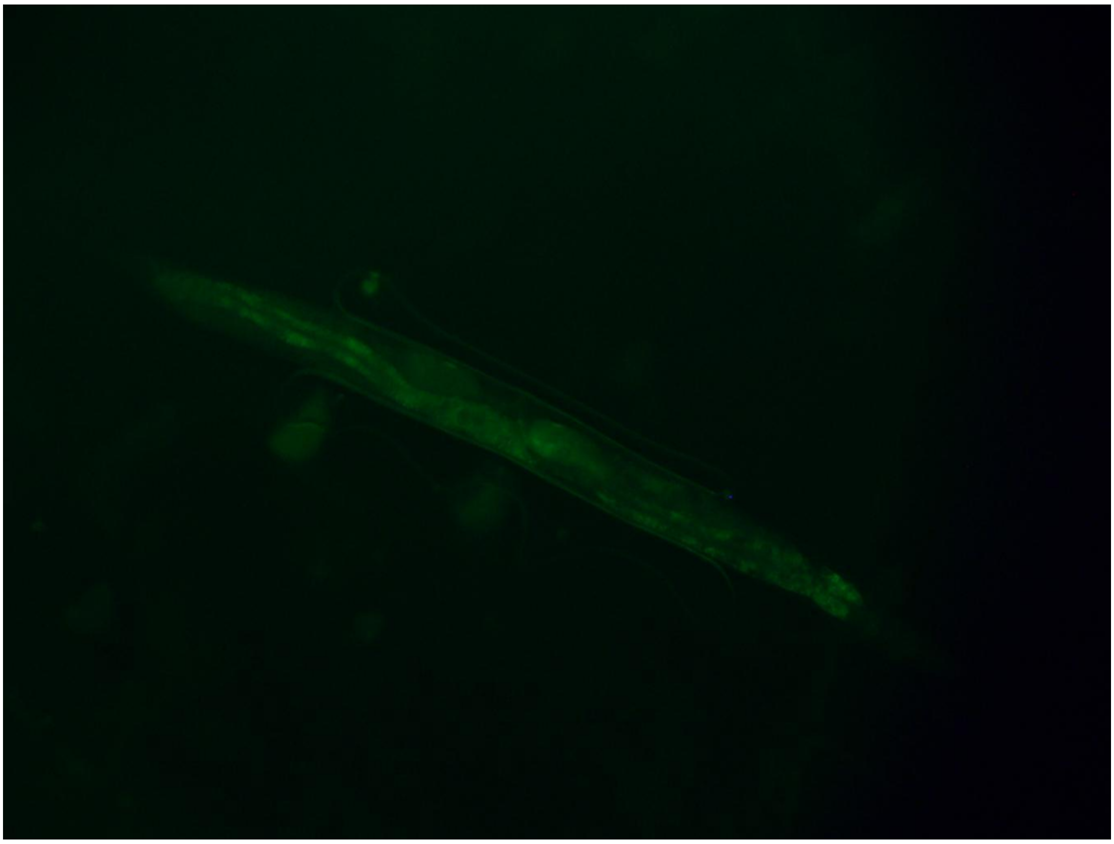

Supplement: S1 Raw images — (ZIP) [file pone.0231972.s006.zip › S1_raw_images.pdf]
